# Complementary antioxidant strategy for diabetic wound healing: myricetin and ceria nanozymes for enhanced reactive oxygen species management

**DOI:** 10.1093/rb/rbag105

**Published:** 2026-06-02

**Authors:** Xiaoxiao Liao, Lei Song, Shancan Wang, Kai Cheng, Ying Xiao, Donghong Li, Shuyan Chen, Yijing Liu, Yinyu Zhao, Xiaolin Zhang, Yuechuan Shen, Menglan He, Rong Wang

**Affiliations:** Marine Science and Technology College, Zhejiang Ocean University, Zhoushan 316022, China; Laboratory of Advanced Theranostic Materials and Technology, Ningbo Institute of Materials Technology and Engineering, Chinese Academy of Sciences, Ningbo 315201, China; Zhejiang International Scientific and Technological Cooperative Base of Biomedical Materials and Technology, Ningbo Cixi Institute of Biomedical Engineering, Ningbo 315300, China; Laboratory of Advanced Theranostic Materials and Technology, Ningbo Institute of Materials Technology and Engineering, Chinese Academy of Sciences, Ningbo 315201, China; Zhejiang International Scientific and Technological Cooperative Base of Biomedical Materials and Technology, Ningbo Cixi Institute of Biomedical Engineering, Ningbo 315300, China; Laboratory of Advanced Theranostic Materials and Technology, Ningbo Institute of Materials Technology and Engineering, Chinese Academy of Sciences, Ningbo 315201, China; Zhejiang International Scientific and Technological Cooperative Base of Biomedical Materials and Technology, Ningbo Cixi Institute of Biomedical Engineering, Ningbo 315300, China; Laboratory of Advanced Theranostic Materials and Technology, Ningbo Institute of Materials Technology and Engineering, Chinese Academy of Sciences, Ningbo 315201, China; Zhejiang International Scientific and Technological Cooperative Base of Biomedical Materials and Technology, Ningbo Cixi Institute of Biomedical Engineering, Ningbo 315300, China; Laboratory of Advanced Theranostic Materials and Technology, Ningbo Institute of Materials Technology and Engineering, Chinese Academy of Sciences, Ningbo 315201, China; Zhejiang International Scientific and Technological Cooperative Base of Biomedical Materials and Technology, Ningbo Cixi Institute of Biomedical Engineering, Ningbo 315300, China; Laboratory of Advanced Theranostic Materials and Technology, Ningbo Institute of Materials Technology and Engineering, Chinese Academy of Sciences, Ningbo 315201, China; Zhejiang International Scientific and Technological Cooperative Base of Biomedical Materials and Technology, Ningbo Cixi Institute of Biomedical Engineering, Ningbo 315300, China; Laboratory of Advanced Theranostic Materials and Technology, Ningbo Institute of Materials Technology and Engineering, Chinese Academy of Sciences, Ningbo 315201, China; Zhejiang International Scientific and Technological Cooperative Base of Biomedical Materials and Technology, Ningbo Cixi Institute of Biomedical Engineering, Ningbo 315300, China; Laboratory of Advanced Theranostic Materials and Technology, Ningbo Institute of Materials Technology and Engineering, Chinese Academy of Sciences, Ningbo 315201, China; Zhejiang International Scientific and Technological Cooperative Base of Biomedical Materials and Technology, Ningbo Cixi Institute of Biomedical Engineering, Ningbo 315300, China; Laboratory of Advanced Theranostic Materials and Technology, Ningbo Institute of Materials Technology and Engineering, Chinese Academy of Sciences, Ningbo 315201, China; Zhejiang International Scientific and Technological Cooperative Base of Biomedical Materials and Technology, Ningbo Cixi Institute of Biomedical Engineering, Ningbo 315300, China; Marine Science and Technology College, Zhejiang Ocean University, Zhoushan 316022, China; Department of Emergency, Zhoushan Hospital of Zhejiang Province, Zhoushan 316021, China; Marine Science and Technology College, Zhejiang Ocean University, Zhoushan 316022, China; Laboratory of Advanced Theranostic Materials and Technology, Ningbo Institute of Materials Technology and Engineering, Chinese Academy of Sciences, Ningbo 315201, China; Zhejiang International Scientific and Technological Cooperative Base of Biomedical Materials and Technology, Ningbo Cixi Institute of Biomedical Engineering, Ningbo 315300, China

**Keywords:** diabetic wound healing, ceria nanozymes, myricetin, complementary antioxidant therapy, reactive oxygen species scavenging

## Abstract

Diabetic wounds are characterized by persistent oxidative stress, hypoxia and chronic inflammation, leading to delayed healing and high risk of recurrence. Conventional antioxidants such as polyphenols can rapidly scavenge reactive oxygen species (ROS) but show short-lived and unstable activity, whereas inorganic nanozymes provide longer-term catalysis but respond more slowly to acute oxidative bursts. Here, a multifunctional hydrogel dressing (PBX@CCM) was constructed by integrating myricetin (MYR)-loaded ceria nanozymes (CCMNRs) into a dynamically crosslinked polyvinyl alcohol/borax/xanthan gum (PBX) network to achieve complementary antioxidant therapy for diabetic wounds. CCMNRs combined the rapid ROS-scavenging ability of MYR with the sustained, auto-regenerative redox cycling of Ce^3+^/Ce^4+^, enabling dual-action antioxidant behavior with multi-enzyme-mimicking activities, efficient ·OH and H_2_O_2_ clearance, oxygen generation and inhibition of advanced glycation end products formation. PBX@CCM hydrogel exhibited a porous structure and self-healing properties, suitable rheological behavior and good cytocompatibility and hemocompatibility. In lipopolysaccharide-stimulated macrophages, PBX@CCM reduced intracellular ROS levels and promoted M1-to-M2 polarization, indicating antioxidant and immunomodulatory effects. In a streptozotocin-induced diabetic rat model with full-thickness wounds, PBX@CCM significantly accelerated wound closure with improved tissue regeneration. These results demonstrate that PBX@CCM hydrogel provides a rapid-and-sustained antioxidant platform with immune regulation, offering a promising strategy for the treatment of diabetic wounds.

## Graphical Abstract

**Graphical Abstract:**
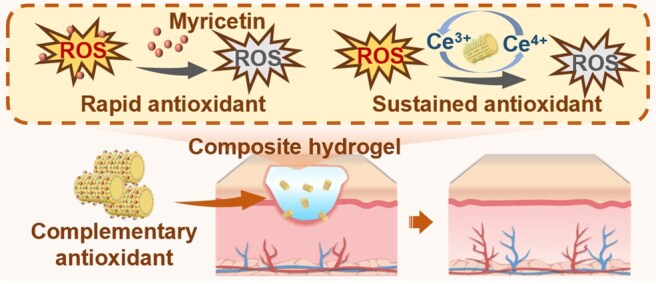

## Introduction

Diabetes mellitus (DM) is a widespread chronic metabolic disease and has become a serious global public health problem. Among its many complications, chronic wounds, especially diabetic foot ulcers, are a major cause of disability, amputation and even mortality [1]. Clinical data indicate that approximately 25% of diabetic patients suffer from delayed or non-healing skin wounds [2]. These wounds are often characterized by high incidence, frequent recurrence and poor treatment outcomes, placing a heavy burden on healthcare systems worldwide [3]. Despite standard wound care, many diabetic ulcers remain in a prolonged inflammatory state with recurrent oxidative insults, leading to repeated tissue damage and poor healing outcomes.

Reactive oxygen species (ROS) play a crucial role in wound healing. Under normal conditions, moderate levels of ROS are generated after injury and participate in host defense and tissue repair [4]. However, in diabetic patients, the prolonged hyperglycemia impairs endogenous antioxidant systems, making wounded tissues prone to excessive ROS accumulation [5]. When ROS levels exceed cellular antioxidant capacity, severe oxidative stress occurs, leading to lipid membrane damage, protein oxidation and nucleic acid injury. This process induces apoptosis of key repair cells such as keratinocytes and fibroblasts and suppresses their proliferation and migration [6]. At the same time, excessive ROS amplify inflammatory signaling, prolong the inflammatory phase and inhibit granulation tissue formation and re-epithelialization [7, 8]. As a result, diabetic wounds experience a cascade of events, with oxidative stress leading to chronic inflammation and ultimately wound healing failure, making effective ROS control a critical target for improving diabetic wound healing.

To address oxidative stress in diabetic wounds, antioxidant-based strategies have been widely explored. Polyphenolic compounds are among the most commonly studied antioxidants due to their strong free radical scavenging ability [9]. They can rapidly neutralize ROS, such as ·OH, ·O_2_^−^ and H_2_O_2_ by interrupting free radical chain reactions, thereby reducing lipid peroxidation, protein damage and DNA injury. Myricetin (MYR), a natural flavonoid found in fruits such as bayberries, strawberries and grapes, is rich in phenolic hydroxyl groups and exhibits notable antioxidant, anti-inflammatory and glucose-lowering effects [10, 11]. These properties make MYR attractive for early-stage oxidative stress regulation in diabetic wounds. However, polyphenolic antioxidants also suffer from inherent limitations. They are easily oxidized or degraded in physiological environments and generally have a short effective duration [12]. Under the fluctuating glucose conditions of diabetes, repeated ROS bursts rapidly consume phenolic hydroxyl groups, leading to antioxidant exhaustion. As a result, these agents often fail to provide sustained protection, leading to rapid scavenging, followed by depletion and subsequent re-injury. This lack of long-term antioxidant capacity greatly limits their effectiveness in chronic wound environments. These features highlight an unmet need for antioxidant systems that can quickly suppress the early oxidative burst while maintaining long-term ROS control under recurrent oxidative stress.

In recent years, nanozymes have emerged as a promising alternative for sustained antioxidant therapy [13]. Among them, cerium oxide (ceria, CeO_2_) nanozymes are of particular interest due to their reversible redox transition between Ce^3+^ and Ce^4+^ states [14, 15]. This redox cycling enables continuous ROS scavenging and regeneration of antioxidant activity. Compared with natural enzymes, ceria nanozymes show higher stability, lower cost and better tolerance to harsh conditions, making them suitable for chronic inflammatory diseases [16]. Nevertheless, monometallic ceria nanozymes often exhibit limited catalytic efficiency in complex biological environments due to slow electron transfer and insufficient oxygen vacancy density [17]. As a result, they are less effective in rapidly reducing severe oxidative stress during the early stages of diabetic wound healing and struggle to meet the dynamic demands of different healing phases when used alone.

**Figure 1:**
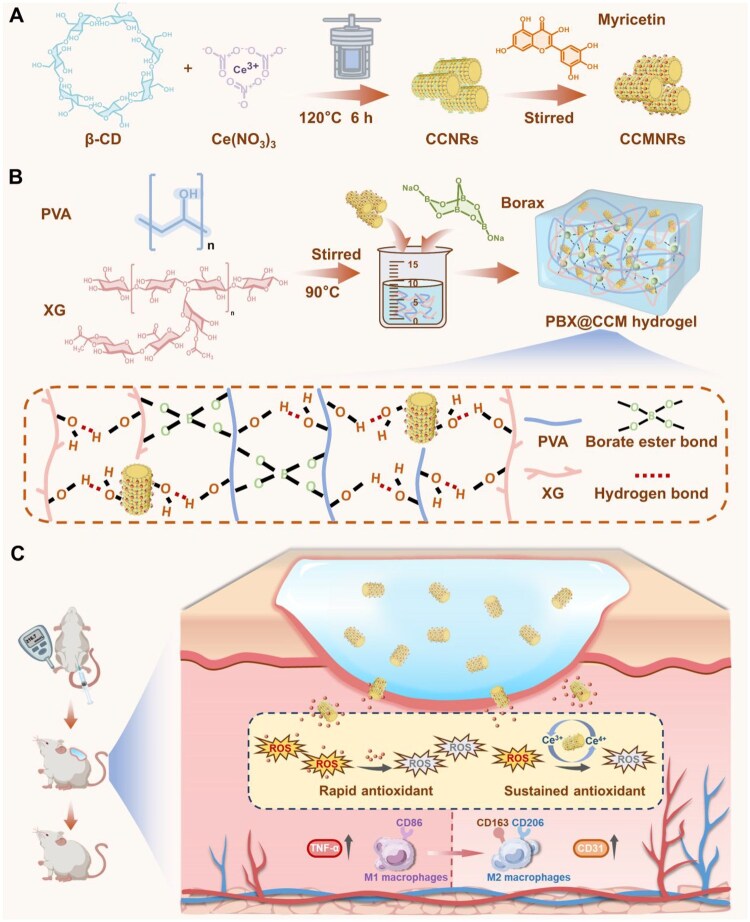
Schematic illustration of PBX@CCM hydrogel as a multifunctional material for diabetic chronic wound healing. (**A**) Synthesis process of CCMNRs. (**B**) Preparation and mechanism of PBX@CCM hydrogel. (**C**) PBX@CCM hydrogel effectively reduces excessive ROS accumulation in wounds through rapid ROS scavenging by MYR and sustained antioxidant effects of ceria. It modulates macrophage polarization, alleviates inflammation and accelerates wound healing.

Based on these considerations, this study proposes a dual antioxidant strategy by combining the rapid ROS scavenging action of MYR with the sustained antioxidant activity of ceria nanozymes for diabetic wound healing. In this staged design, MYR addresses the acute ROS surge immediately after injury, whereas ceria nanozymes sustain ROS clearance through Ce^3+^/Ce^4+^ redox cycling, together providing continuous protection across distinct healing phases. While ceria nanozymes have been widely studied for their sustained ROS scavenging and wound-healing functions [18], and MYR exhibits strong short-term antioxidant effects, their integration into a staged antioxidant system for chronic diabetic wounds has not been systematically investigated. To achieve this, β-cyclodextrin (β-CD) was employed as a carrier to integrate MYR and ceria nanozyme into a composite system (CCMNRs) (Figure 1A). β-CD was anchored onto the ceria surface through hydrogen bonding and electrostatic interactions [19], while MYR was loaded via host–guest interactions and coordination bonding [20, 21]. In this system, MYR rapidly neutralizes ROS during the early inflammatory phase, while ceria nanozymes provide sustained ROS clearance through their redox cycling ability, ensuring sustained protection and overcoming the short-lived effects of traditional antioxidants.

To enable wound-adaptable delivery, the composite nanozyme system was incorporated into a multifunctional hydrogel (PBX hydrogel) for diabetic wound treatment (Figure 1B), improving local retention and stability at the wound site. The hydrogel was constructed from polyvinyl alcohol (PVA), borax and xanthan gum (XG), forming a dynamic 3D network through reversible borate ester bonds and hydrogen bonding [22]. This structure endows the hydrogel with good biocompatibility, moisture retention and self-healing ability, allowing it to conform closely to irregular wound surfaces (Figure 1C). Moreover, loading composite nanozymes into the hydrogel (denoted as PBX@CCM) imparts multiple enzyme-like activities, including peroxidase (POD)-, superoxide dismutase (SOD)- and catalase (CAT)-mimicking functions, as well as hydroxyl radical scavenging capacity. Overall, PBX@CCM hydrogel is designed to deliver rapid-and-sustained antioxidant therapy with concurrent immune regulation, thereby promoting efficient repair of diabetic wounds.

## Materials and methods

### Materials

Cerium nitrate hexahydrate was purchased from Sigma-Aldrich. MYR, XG, 2,2′-azino-bis(3-ethylbenzothiazoline-6-sulfonic acid) diammonium salt (ABTS), 3,3′,5,5′-tetramethylbenzidine (TMB) and 5,5-dimethyl-1-pyrroline *N*-oxide (DMPO) were obtained from Aladdin Biochemical Technology. β-Cyclodextrin (β-CD), PVA and sodium tetraborate decahydrate (borax) were provided by Sinopharm. Lipopolysaccharide (LPS), and 2,2-diphenyl-1-picrylhydrazyl (DPPH) were purchased from Macklin Biochemical Technology. ROS assay kit with DCFH-DA, Cell Counting Kit-8 (CCK-8) assay, Calcein AM/Propidium Iodide assay kit and 2-(4-amidinophenyl)-6-indolecarbamidine dihydrochloride (DAPI) staining solution were purchased from Beyotime Biotechnology. Streptozocin (STZ) and superoxide dismutase (SOD) activity assay kit were purchased from Solarbio Life Sciences. Dulbecco’s modified Eagle’s medium (DMEM) and penicillin–streptomycin were provided by Gibco. L929 fibroblast cells and RAW 264.7 cells were obtained from National Collection of Authenticated Cell Cultures, Chinese Academy of Sciences.

### Synthesis of CCNRs and CCMNRs

CCNRs were synthesized via a one-pot hydrothermal method according to a previous report [23]. Briefly, 1 mmol of cerium nitrate hexahydrate and 1 mmol of β-CD were dissolved in 15 mL of ultrapure water at room temperature. Subsequently, 0.6 g of NaOH was gradually added under continuous stirring. After 10 min, the mixture was transferred to a Teflon-lined stainless-steel autoclave and heated at 120°C for 6 h, then allowed to cool to room temperature. The precipitate was collected by centrifugation and washed alternately with 2% NaCl solution and ultrapure water to remove ionic residues, yielding CCNRs. Then 25 mg of MYR was added to the CCNRs suspension and stirred for 6 h. The resulting CCMNRs were collected by centrifugation and finally dispersed in water for subsequent use. For comparison, CNRs were synthesized using the same procedure without the addition of β-CD.

### Characterization of CCNRs and CCMNRs

The morphology of CCNRs and CCMNRs was examined using transmission electron microscopy (TEM, FEI Talos F200X), selected area electron diffraction (SAED) and X-ray diffraction (XRD, Bruker D8 Advance Davinci). Elemental composition and distribution were characterized by energy-dispersive X-ray spectroscopy (EDS) coupled with TEM. Hydrodynamic diameter and zeta potential were determined using dynamic light scattering (DLS, Anton Paar Litesizer 500). The cerium content was measured via inductively coupled plasma mass spectrometry (ICP-MS, Agilent 7850). The β-CD content in the composite nanozymes, as well as the actual loading efficiency (ratio of the mass of MYR loaded in CCMNRs to the initial mass of MYR added) and release behavior of MYR, was measured using ultraviolet–visible spectrophotometry (Agilent Cary 5000). X-ray photoelectron spectroscopy (XPS, AXIS SUPRA+) was used to acquire spectra of CNRs, CCNRs, CCMNRs, β-CD and MYR. Fourier transform infrared spectroscopy (FT-IR, Thermo Fisher IS 50) was employed to confirm the formation of the CCMNRs inclusion complex.

### Preparation of PBX@CCM hydrogel

PVA was dissolved in 15 mL of ultrapure water and heated at 90°C under stirring for 1 h to obtain a PVA solution (10 wt.%). Next, XG (1 wt.% in final solution) and CCMNRs (3 mg/mL in final solution) were added to the PVA solution. The mixture was stirred at 60°C for 1 h to obtain a homogeneous solution. Subsequently, 5 mL of borax solution (1.6 wt.%) was added and gently stirred to form the PBX@CCM hydrogel. For comparison, the PB hydrogel was prepared using the same procedure but without the addition of XG or CCMNRs; the PBX hydrogel was prepared without CCMNRs; the PBX@MYR hydrogel was prepared by replacing CCMNRs with free MYR (0.23 mg/mL) and the PBX@CC hydrogel was prepared by replacing CCMNRs with CCNRs (2.77 mg/mL).

### Characterization of PBX@CCM hydrogel

Scanning electron microscopy (SEM, Hitachi Regulus 8230) was employed to observe the microstructure and internal architecture of PB, PBX and PBX@CCM hydrogels. The hydrogels were cryo-frozen in liquid nitrogen, followed by freeze-drying. The dried samples were sputter-coated with gold prior to imaging. The distribution of Ce within the PBX@CCM hydrogel was analysed using EDS. FT-IR spectroscopy was performed to characterize the hydrogels. The rheological properties and self-healing behavior of the PBX@CCM hydrogel were evaluated using a rotational rheometer (WATERS HR-2).

For *in vitro* degradation testing, 2 g of hydrogel samples were immersed in 30 mL phosphate-buffered saline (PBS, 10 mM, pH 7.4) at 37°C. At predetermined time intervals, the samples were removed, gently blotted to remove surface moisture and weighed. The residual weight was calculated using Equation (1):


(1) $$\mathrm{Mass}\;\mathrm{remaining}(\%)=W_{t}/W_{0}\times 100\%$$

where *W_t_* and *W*_0_ represent the weight of the hydrogel at time *t* and the initial weight, respectively.

*In vitro* drug release experiments were conducted under conditions simulating the physiological environment (PBS buffer, pH 7.4, 37°C). Five grams of PBX@CCM hydrogel were placed in a 12–14 kDa dialysis bag and immersed in 500 mL of PBS solution. At predetermined time intervals, aliquots were collected. The concentration of MYR in the dialysate was determined using ultraviolet–visible spectrophotometry at 254 nm to plot the drug release curve.

### Antioxidant capacity evaluation

To evaluate the multi-enzyme-mimicking antioxidant activity of the nanozymes and hydrogels, standard antioxidant assays were conducted, including DPPH and ABTS radical scavenging tests, as well as POD-, SOD- and CAT-like activity assays. For the DPPH assay, 2 mL of 0.1 mM DPPH solution was mixed with 100 µL of nanozyme dispersion or 100 mg of hydrogel. The mixture was incubated in the dark for 30 min, and the absorbance at 517 nm was recorded to calculate the DPPH radical scavenging efficiency. For the ABTS assay, an ABTS working solution with an absorbance of 0.70 ± 0.02 was prepared. Then, 100 µL of nanozyme dispersion or 100 mg of hydrogel was added to 2 mL of ABTS solution, followed by incubation in the dark for 30 min. The absorbance at 734 nm was measured, and the ABTS scavenging efficiency was calculated.

POD-like activity was assessed using TMB as the substrate. Briefly, CCNRs, CCMNRs or hydrogel samples at different concentrations were mixed with TMB in sodium acetate buffer (pH 4.5) and incubated at room temperature for 30 min. The formation of oxidized TMB was quantified by measuring the absorbance at 652 nm. SOD-like activity of the nanozymes and hydrogels was evaluated using a WST-1 assay kit by recording the absorbance at 450 nm. CAT-like activity was assessed by monitoring both oxygen generation and H_2_O_2_ consumption. Nanozymes or hydrogels were mixed with 10 mM H_2_O_2_, and dissolved oxygen was measured every 20 min for 4 h using a dissolved oxygen meter (Leici JPBJ-608). In parallel, residual H_2_O_2_ was quantified using titanium sulfate (Ti(SO_4_)_2_), based on the characteristic absorbance at 415 nm, to further confirm H_2_O_2_ consumption.

Raman spectroscopy was performed to investigate the redox-related behavior of CCMNRs. Raman spectra were collected at 0, 1 and 12 h for 10 mg of CCMNRs and for a mixture of 10 mg of CCMNRs with 0.1 mL of 10% H_2_O_2_ under 488 nm laser excitation [19]. The auto-regenerative interconversion between Ce^3+^ and Ce^4+^ in CNRs, CCNRs and CCMNRs was analysed using XPS. Specifically, 10 mg of each sample was analysed directly, and an additional set of samples (10 mg) was treated with 0.15 mM H_2_O_2_. XPS spectra were recorded on day 0 and day 6 for comparison.

The radical scavenging capacity of CCNRs and CCMNRs was further evaluated by electron spin resonance (ESR) using DMPO as a spin trap. In a typical procedure, 100 µL of CCMNRs dispersion (2.5, 5 or 10 mM) was added to a mixture containing 50 µL of 1 mM FeSO_4_ and 10 µL of 1 M DMPO. The reaction was initiated by adding 50 µL of 10 mM H_2_O_2_. After 5 min, the reaction mixture was transferred into a capillary tube for ESR measurement. A color change experiment was conducted to visually demonstrate the response of CCNRs, CCMNRs, PBX@CC and PBX@CCM hydrogels to repeated oxidative challenges. Briefly, 1 mM H_2_O_2_ was added to each sample, and photographs were taken to document color changes. After the samples had returned to their initial color, 1 mM H_2_O_2_ was added again and images were acquired to record the response after the second exposure.

In the cyclic DPPH scavenging assay, 2 mL of 0.1 mM DPPH solution was mixed with 100 µL of nanozyme dispersion, incubated under dark conditions for 30 min, followed by centrifugation to obtain the supernatant and the absorbance at 517 nm was recorded. After each cycle, the supernatant was discarded and replaced with 2 mL of fresh DPPH solution. The incubation and absorbance measurements were repeated for a total of six consecutive cycles, and the DPPH free radical scavenging efficiency for each cycle was calculated.

### MYR antioxidant stability analysis

To evaluate the antioxidant stability of MYR, MYR powder was dissolved in 10 mL of anhydrous ethanol to obtain a 100 µg/mL MYR solution, which was then incubated either in the dark at its original pH, in the dark after adjustment to pH 5.0 or 9.0, or under ultraviolet irradiation at 254 nm for 6 h. After treatment, the radical scavenging activity was measured by the ABTS assay as described above, to assess the stability of the MYR antioxidant activity under different pH and light conditions.

### Glycation inhibition ability evaluation

Advanced glycation end products (AGEs) formation in the presence or absence of hydrogels was evaluated according to a previously reported method with minor modifications [24]. A glycation reaction solution was prepared by dissolving bovine serum albumin (BSA, 20 mg/mL) and glucose (100 mg/mL) separately in 10 mM PBS, filtered each through a 0.22 µm membrane and then mixing them in equal volume. Glycation mixtures (10 mL) were prepared with or without hydrogel (1 g), and the corresponding PBS controls (with or without hydrogel) were prepared in parallel. After incubation at 37°C for 5 days, the samples were cooled to room temperature. Aliquots (200 µL) were transferred to a 96-well plate, and fluorescence was measured using a microplate reader with an excitation wavelength at 370 nm and an emission wavelength at 440 nm. The inhibition ratio of AGEs was calculated according to Equation (2):


(2) AGEs inhibition ratio (%)=[1-(A-B)/(C-D)]×100%

where *A* represents the fluorescence intensity of the glycated system containing hydrogel; *B* represents the fluorescence intensity of the PBS solution containing hydrogel; *C* represents the fluorescence intensity of the glycated system without hydrogel and *D* represents the fluorescence intensity of the PBS solution without hydrogel.

### *In vitro* biocompatibility assessment

CCK-8 assays were performed to evaluate *in vitro* cytotoxicity. For preparation of hydrogel extracts, ultraviolet-sterilized hydrogels (200 mg) were incubated in 2 mL culture medium at 37°C for 24 h. L929 cells were seeded in 96-well plates at a density of 1 × 10^4^ cells per well and cultured overnight. The medium was then replaced with CCMNRs at varied concentrations or with different hydrogel extracts, and the cells were incubated for 1, 3 and 5 days. Fresh culture medium and an aqueous solution containing 1 wt.% of zinc diethyldithiocarbamate served as the negative and positive controls, respectively. At each time point, 100 µL of fresh medium containing 10 µL of CCK-8 reagent was added to each well according to the manufacturer’s protocol. After incubation, the absorbance at 450 nm was measured using a microplate reader.

For the live–dead staining assay, L929 cells were seeded in glass-bottom confocal dishes at a density of 1 × 10^5^ cells per dish and cultured overnight. The cells were then incubated with different hydrogel extracts for 1, 3 and 5 days. At each time point, the medium was replaced with Calcein AM/Propidium Iodide staining solution and incubated for 30 min. The stained cells were imaged using a confocal laser scanning microscope.

A hemolysis assay was conducted to evaluate blood compatibility. Equal volumes of CCMNRs at varied concentrations or different hydrogel extracts were mixed with a 2% rat red blood cell (RBC) suspension and incubated at 37°C for 2 h. The mixtures were gently inverted twice every 30 min to ensure sufficient contact between RBCs and the samples. After incubation, the suspensions were centrifuged at 2000 rpm for 10 min. Photographs were taken, and the absorbance of the supernatants was measured at 545 nm. Physiological saline was used as the negative control, and 2% Triton X-100 was used as the positive control. The hemolysis ratio was calculated according to Equation (3):


(3) $$\mathrm{Hemolysis}\;\mathrm{ratio}(\%)=(D_{s}-D_{n})/(D_{w}-D_{n})\times 100\%$$

where *D_s_*, *D_n_* and *D_w_* represent the absorbance of the samples, physiological saline, and Triton X-100 groups, respectively.

### Intracellular ROS scavenging capacity of PBX@CCM hydrogel

RAW 264.7 cells were seeded in confocal culture dishes at a density of 5 × 10^5^ cells per dish and cultured at 37°C in a humidified atmosphere containing 5% CO_2_ for 24 h. The cells were then co-treated with LPS (5 µg/mL) or H_2_O_2_ (600 µM) and either PBX hydrogel extract, PBX@MYR hydrogel extract or PBX@CCM hydrogel extract for an additional 24 h. After treatment, the cells were incubated with DCFH-DA probe (10 µM) for 30 min. The intracellular ROS level was evaluated by recording the fluorescence intensity using a confocal laser scanning microscope.

### Immunomodulatory capacity of PBX@CCM hydrogel

RAW 264.7 cells were cultured and stimulated with LPS (5 µg/mL) in the presence of PBX, PBX@MYR or PBX@CCM hydrogel extracts as described above. After treatment, the cells were fixed with 4% paraformaldehyde for 30 min, washed three times with PBS and blocked with 5% goat serum at room temperature for 1 h. Primary antibodies against CD86, CD163 and CD206 were diluted in blocking buffer according to the manufacturer’s instructions and incubated with the cells overnight at 4°C in the dark. The cells were then washed three times with PBS and incubated with the corresponding secondary antibodies for 2 h at room temperature in the dark. Nuclei were counterstained with DAPI for 20 min. After washing three times with PBS, fluorescence images were acquired using a confocal laser scanning microscope. The antibodies used are listed in Supplementary Table S1.

After 24 h of LPS stimulation, cell culture supernatants from each group were collected and centrifuged at 1000 rpm for 10 min. The concentrations of TNF-α, IL-6, IL-10 and TGF-β1 in the supernatants were quantified using commercial ELISA kits (Solarbio) according to the manufacturer’s instructions. Absorbance was measured at 450 nm, and cytokine concentrations were calculated from standard curves and expressed in pg/mL.

### *In vivo* evaluation of diabetic wound healing

All animal experiments were conducted in accordance with the Guide for the Care and Use of Laboratory Animals (National Research Council) and the Laboratory Animal—Guidelines for Ethical Review of Animal Welfare (GB/T 35892-2018). The protocol was approved by Zhejiang Huitong Test & Evaluation Technology Group Co., Ltd IACUC Committee (approval number: HT-2025-LWFB-0058). Male Sprague-Dawley rats weighing 220–250 g were used in this study. After 1 week of acclimation, diabetes was induced by intraperitoneal injection of STZ (40 mg/kg) for three consecutive days. Successful induction was confirmed by non-fasting blood glucose levels ≥16.7 mM and stable hyperglycemia for one week. The animals were randomly assigned to three groups (*n* = 8 per group): control, PBX and PBX@CCM. All procedures were performed under isoflurane anesthesia. After diabetes confirmation, the dorsal fur was shaved, and the skin was cleaned and disinfected. A full-thickness excisional wound (8 mm in diameter) was created on the dorsum. To minimize wound contraction, a circular silicone ring (inner diameter of 12 mm) was fixed around the wound. Subsequently, 500 µL of saline (control), PBX hydrogel or PBX@CCM hydrogel was applied to the wound surface. The wounds were then covered with a 3M Tegaderm film to prevent scratching or biting until the end of the experiment. The hydrogels were replaced on days 3, 7 and 10, respectively. Digital photographs were taken on days 0, 3, 7, 10 and 14. Wound areas were quantified using ImageJ software, and the wound healing ratio was calculated according to Equation (4):


(4) $$\mathrm{Wound}\;\mathrm{healing}\;\mathrm{ratio}(\%)=(A_{0}-A_{t})/A_{0}\times 100\%$$

where *A*_0_ is the initial wound area and *A_t_* is the wound area at the indicated time point.

To evaluate the therapeutic efficacy of PBX@CCM hydrogel at different stages of diabetic wound healing, rats were euthanized on days 7 and 14, and wound tissues were harvested. The collected skin samples were fixed with 4% paraformaldehyde and embedded in paraffin. Hematoxylin and eosin (H&E) staining was performed to assess histological regeneration, and Masson’s trichrome staining was used to evaluate collagen deposition. Immunohistochemical staining for CD31 and TNF-α was performed to evaluate angiogenesis and inflammation during wound healing stages. Stained sections were imaged using a fluorescence microscope (Leica DMIL LED Fluo). Quantitative analysis of the staining results was conducted using ImageJ software. The antibodies used are listed in Supplementary Table S1.

### Statistical analysis

All tests were conducted in at least three replicates. Quantitative data are expressed as mean ± standard deviation. Statistical analysis was performed using Origin software. Intergroup differences were assessed using one-way analysis of variance (ANOVA), while differences between two groups were analysed using a Student’s *t*-test. A value of *P* < 0.05 was considered statistically significant. n.s. indicates non-significant, * *P* < 0.05, ** *P* < 0.01 and *** *P* < 0.001.

## Results and discussion

### Synthesis and characterization of CCMNRs

**Figure 2:**
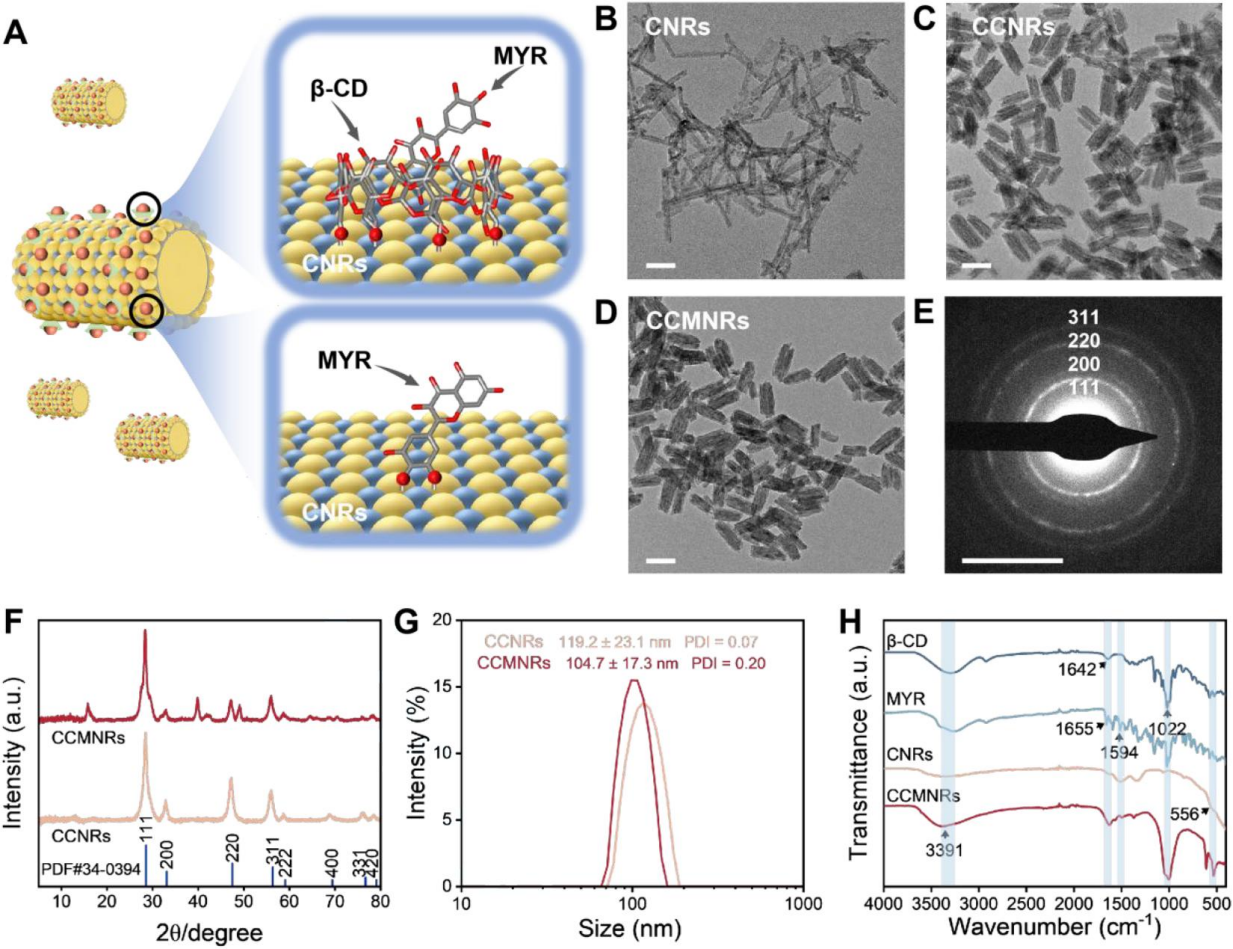
Preparation and characterization of CCMNRs. (**A**) Simulated surface structures of CCMNRs. TEM images of (**B**) CNRs, (**C**) CCNRs and (**D**) CCMNRs. Scale bar = 50 nm. (**E**) SAED images of CCMNRs. Scale bar = 5 nm^−1^. (**F**) XRD analysis of CCNRs and CCMNRs. (**G**) Hydrodynamic diameter distributions of CCNRs and CCMNRs. (**H**) FT-IR spectra of β-CD, MYR, CNRs and CCMNRs.

CCMNRs were synthesized as illustrated in Figure 1A. Briefly, β-CD-coated nanoceria (CCNRs) were first prepared via a hydrothermal method [19], and MYR was subsequently loaded through host–guest interactions with β-CD and coordination interactions with the ceria surface to form CCMNRs (Figure 2A) [25, 26]. TEM showed that CNRs (synthesized using only cerium nitrate hexahydrate, Figure 2B) exhibited an irregular rod-like morphology with lengths of approximately 100–200 nm. In contrast, both CCNRs (synthesized from cerium nitrate hexahydrate and β-CD, Figure 2C) and CCMNRs (further loaded with MYR, Figure 2D) displayed uniform, short rod-like structures with lengths of about 50–70 nm, indicating that β-CD coating effectively suppressed excessive nanorod growth. The corresponding EDS elemental mapping images revealed that Ce and O signals were concentrated in the nanorod regions, consistent with the TEM morphology, verifying the successful synthesis of the CCMNRs (Supplementary Figure S1). SAED analysis confirmed a well-defined crystalline structure of CCMNRs (Figure 2E). XRD analysis of CCNRs and CCMNRs revealed diffraction peaks at 2*θ* values of 28.5°, 33.1°, 47.5°, 56.3°, 59.1°, 69.4°, 76.7° and 79.1°, corresponding to the (1 1 1), (2 0 0), (2 2 0), (3 1 1), (2 2 2), (4 0 0), (3 3 1) and (4 2 0) planes of the cubic fluorite structure of ceria nanozyme (Figure 2F), confirming that both CCNRs and CCMNRs retained the typical crystal structure [27]. DLS analysis revealed hydrodynamic diameters for CCNRs and CCMNRs, measured as 119.2 ± 23.1 and 104.7 ± 17.3 nm, respectively (Figure 2G). These values were larger than the geometric sizes observed by TEM, which is expected because DLS reports the apparent hydrodynamic diameter of particles surrounded by a hydrated and ligand shell, whereas TEM primarily reflects the dry solid core. Zeta potential measurements indicated that β-CD increased the absolute zeta potential of ceria, likely due to its binding to the nanoparticle surface via hydrogen bonding and introducing additional negative charges. Subsequent loading of MYR decreased the absolute zeta potential, which can be ascribed to host–guest complex formation between MYR and β-CD and partial shielding of surface charges (Supplementary Figure S2). FT-IR and XPS analyses confirmed the successful incorporation of β-CD and MYR. In the FT-IR spectrum of CCMNRs (Figure 2H), characteristic Ce–O vibrations of nanoceria were observed at 556 cm^−1^, along with β-CD-associated peaks at 1022 cm^−1^ (C–O–C) [28]. In addition, MYR-related peaks appeared at 1655 cm^−1^ (C=O) and 1594 cm^−1^ (aromatic C=C stretching) [29], together with a broad O–H stretching band at 3391 cm^−1^. Comparison with the spectra of β-CD and MYR allowed straightforward assignment of these features, confirming their presence in CCMNRs. XPS analysis of the C 1s and O 1s spectra revealed interactions among β-CD, MYR and CCMNRs (Supplementary Figure S3). The C 1s and O 1s spectra of CCMNRs exhibited characteristic peaks of β-CD and MYR, with all the corresponding peaks shifted toward higher binding energies compared with the pure compounds, indicating changes in their chemical environments due to interaction with the ceria surface. In addition, the O 1s spectrum of CCMNRs showed a lattice oxygen peak from ceria, confirming the presence of the ceria core. Taken together, these features suggest that β-CD and MYR are integrated into the CCMNRs nanostructure through interfacial interactions rather than being simply physically mixed.

To further elucidate the composition of CCMNRs, the ceria content was quantified using ICP-MS. The actual MYR loading efficiency was determined using an ultraviolet–visible calibration curve based on the absorbance of MYR at 254 nm (Supplementary Figure S4). Calculations reveal that the loading efficiency of MYR in CCMNRs is 96.88 ± 0.40%. The β-CD content was calculated from the amount of MYR displaced by amantadine [30]. On this basis, the mass percentages of the main components in CCMNRs were determined to be ceria: β-CD:MYR = 83.48:8.75:7.77. The release behavior of MYR from CCMNRs was then evaluated *in vitro*. CCMNRs showed a sustained MYR release profile for up to 32 h (Supplementary Figure S5), which can be attributed to the combined effects of the β-CD host-guest interaction [31] and coordination between MYR and ceria [32]. This controlled release is expected to prolong the antioxidant action of MYR at the wound site after administration.

### Antioxidant and auto-regenerative ROS scavenging performance of CCNRs and CCMNRs

**Figure 3:**
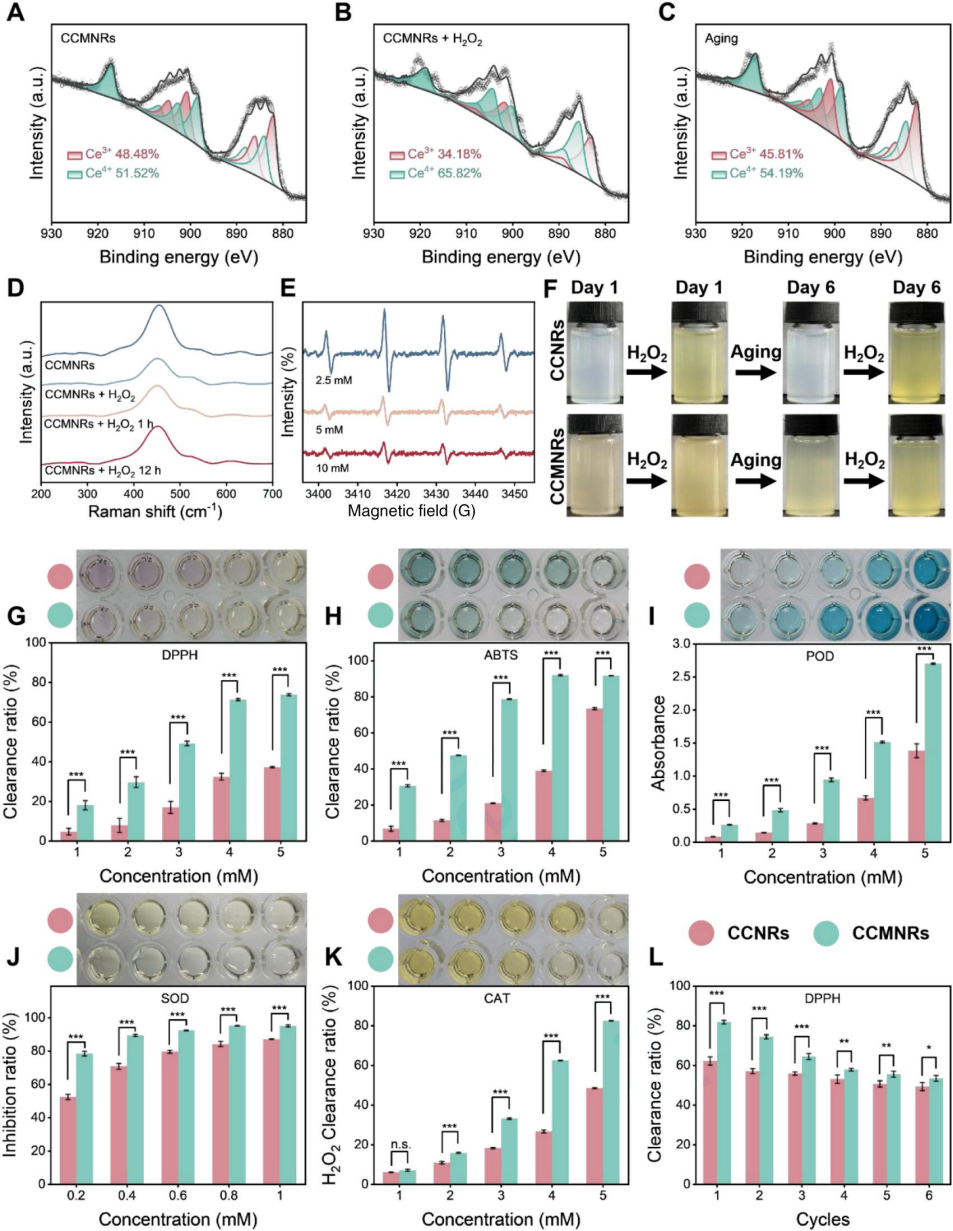
Auto-regenerative ROS scavenging properties of CCNRs and CCMNRs. XPS Ce 3d core-level spectra of (**A**) as-prepared CCMNRs, (**B**) CCMNRs after H_2_O_2_ treatment and (**C**) CCMNRs after H_2_O_2_ treatment followed by aging for 6 days. (**D**) Raman spectra of as-prepared CCMNRs and CCMNRs after addition of H_2_O_2_ recorded at 0, 1 and 12 h. (**E**) ESR spectra of ·OH generated in the Fenton reaction in the presence of CCMNRs at 2.5, 5 and 10 mM. (**F**) Digital photographs showing the color changes of 5 mM CCNRs and CCMNRs dispersions during cyclic 1 mM H_2_O_2_ treatment and subsequent aging. (**G–K**) DPPH and ABTS radical scavenging capacities, and POD-, SOD- and CAT-like enzyme activities of CCNRs and CCMNRs (*n* = 3), with representative images of the corresponding reaction dispersions. (**L**) DPPH radical scavenging performance of CCNRs and CCMNRs over six consecutive cycles (*n* = 3).

MYR provides rapid ROS scavenging, quickly neutralizing ROS in the early stages of the healing process. However, its antioxidant activity is unstable and readily attenuated by factors such as pH and ultraviolet light irradiation, leading to a gradual loss of efficacy (Supplementary Figure S6) [33]. In contrast, the auto-regenerative ROS scavenging properties of nanoceria originate from the reversible redox cycling between Ce^3+^ and Ce^4+^ on the particle surface, which endows CCMNRs with sustained ROS scavenging ability over time [34]. When β-CD and subsequently MYR were integrated with ceria, the XPS Ce 3d spectra of CNRs, CCNRs and CCMNRs show a progressive increase in the Ce^3+^ (882.2, 886.1, 900.8 and 904.7 eV)/Ce^4+^ (883.9, 888.2, 898.4, 902.6, 906.8 and 917.0 eV) ratio (Figure 3A and Supplementary Figure S7). This trend can be attributed to the interactions between β-CD and surface hydroxyl groups on ceria, which promote the formation of oxygen vacancies and thereby facilitate the reduction of Ce^4+^ to Ce^3+^. In addition, MYR, with its strong reducing ability, further converts Ce^4+^ to Ce^3+^, leading to an increased Ce^3+^/Ce^4+^ valence ratio on the ceria surface. XPS was also used to examine the oxidation states of cerium in CCMNRs and their oxidized products, confirming the regeneration behavior of CCMNRs [35]. Quantitative XPS analysis revealed that CCMNRs contained a substantial fraction of Ce^3+^, which is essential for ROS scavenging. As shown in Figure 3A, the Ce^3+^ components accounted for 48.48% of the total cerium, while the Ce^4+^ components represented 51.52% [19]. Upon the addition of H_2_O_2_, the Ce^3+^ fraction decreased to 34.18% (a reduction of about 14.3%, Figure 3B), indicating oxidation of Ce^3+^ to Ce^4+^. Notably, after standing for 6 days, the Ce^3+^ content recovered to 45.81% (Figure 3C), demonstrating that CCMNRs can partially restore their reduced state over time, providing a sustained ROS scavenging capability that lasts throughout the wound healing process.

To further track the redox behavior of CCMNRs, *in situ* Raman spectra were recorded during the oxidation process under 488 nm laser excitation (Figure 3D). A dominant band at around 460 cm^−1^, which is a characteristic Raman peak of crystalline CeO_2_, arises from the symmetric vibration of oxygen atoms around Ce ions and is sensitive to the order of the oxygen lattice [19]. Upon the addition of H_2_O_2_, the signal intensity decreased, suggesting increased defect concentration and partial disorder in the oxygen sublattice. Interestingly, the signal gradually recovered within 1 h and continued to increase over 12 h, indicating that CCMNRs possess regenerative structural stability and redox self-recovery capability under oxidative conditions. The ·OH scavenging ability of CCMNRs was evaluated via ESR spectroscopy (Figure 3E). A characteristic 1:2:2:1 quartet corresponding to the DMPO-OH adduct formed in the Fenton reaction was observed, and the signal intensity decreased in a CCMNRs concentration-dependent manner, indicating efficient ·OH scavenging. Consistent with the spectroscopic observations, visual color changes were also observed. Following H_2_O_2_ treatment, the dispersion of CCNRs changed from nearly white to yellow, while the dispersion of CCMNRs shifted from light brown to brownish-yellow due to the intrinsic color of MYR. Both gradually reverted to their initial colors upon standing (Figure 3F), visually reflecting the reversible redox transition between Ce^3+^ to Ce^4+^. Together, these results indicate that CCMNRs maintain a high capacity for active site regeneration under repeated oxidative challenges, underscoring their sustained ROS scavenging performance.

These are further complemented by multi-enzyme-mimicking activities, including POD-, SOD- and CAT-like functions [36]. Both CCNRs and CCMNRs effectively decomposed radical oxidants (O_2_^−^· and ·OH) as well as non-radical oxidants (H_2_O_2_) in a concentration-dependent manner, as demonstrated in the DPPH (Figure 3G), ABTS (Figure 3H), POD (Figure 3I), SOD (Figure 3J) and CAT (Figure 3K) assays. Importantly, MYR loading further enhanced the multi-enzyme-mimicking activity of CCNRs, highlighting the complementary antioxidant effect between MYR and ceria nanozymes. To directly verify the staged antioxidant mechanism, we conducted cyclic DPPH scavenging assays on CCNRs and CCMNRs (Figure 3L). The DPPH scavenging efficiency of CCNRs decreased gradually with increasing cycles, dropping from 62.2% in Cycle 1 to 49.4% in Cycle 6, reflecting the sustained catalytic antioxidant behavior of the ceria nanozyme. In contrast, CCMNRs exhibited a biphasic decay pattern. During the first three cycles, the scavenging rate dropped sharply from 81.8% to 64.5%, attributable to the progressive consumption of MYR’s phenolic hydroxyl groups through irreversible radical scavenging. From Cycles 4 to 6, the decline slowed markedly (only 5%), and the decay trend converged with that of CCNRs, indicating that MYR’s contribution had been largely exhausted and the catalytic activity of the ceria nanozyme had become the dominant antioxidant source. This distinct biphasic profile provides direct experimental evidence for the staged antioxidant design: MYR delivers rapid, stoichiometric ROS scavenging in the early phase, while ceria nanozymes ensure sustained, catalytic ROS clearance in the later phase. Together, they form a complementary dual-action antioxidant mechanism. Overall, these results confirm that MYR and CCNRs act complementarily, and that CCMNRs combine strong antioxidant activity with robust auto-regenerative ROS scavenging capability.

Finally, the biocompatibility of CCMNRs was assessed to ensure their suitability for biomedical applications. CCK-8 assays (Supplementary Figure S8) and hemolysis tests (Supplementary Figure S9) showed that at the CCMNRs concentrations of ≤ 3 mg/mL, cell viability remained above 80% and hemolysis ratios were consistently below 5%. These findings indicate that CCMNRs possess acceptable cytocompatibility and blood compatibility within the tested concentration range.

### Synthesis and characterization of PBX@CCM hydrogel

To translate the rapid-and-sustained complementary antioxidant activity of CCMNRs into a wound-applicable form, the nanorods were incorporated into a dynamically crosslinked, injectable hydrogel matrix. Compared with direct administration of nanoparticle dispersions, a hydrogel dressing can better retain CCMNRs at the wound site, provide a moist microenvironment and match the irregular shape and dynamic movement of chronic wounds. The PBX@CCM hydrogel was prepared through chemical and physical crosslinking (Figure 1B). Since CCMNRs at a concentration of 3 mg/mL exhibited good biocompatibility (Supplementary Figures S8 and S9), this concentration was selected for incorporation into the hydrogel preparation. XG and CCMNRs were mixed with borax in a PVA solution, forming borate ester bonds between PVA, XG and borax and thereby generating a 3D hydrogel network. Additional hydrogen bonding between the hydroxyl groups of PVA and XG further strengthened the network, ultimately yielding a self-healing composite hydrogel (PBX@CCM) loaded with CCMNRs.

**Figure 4:**
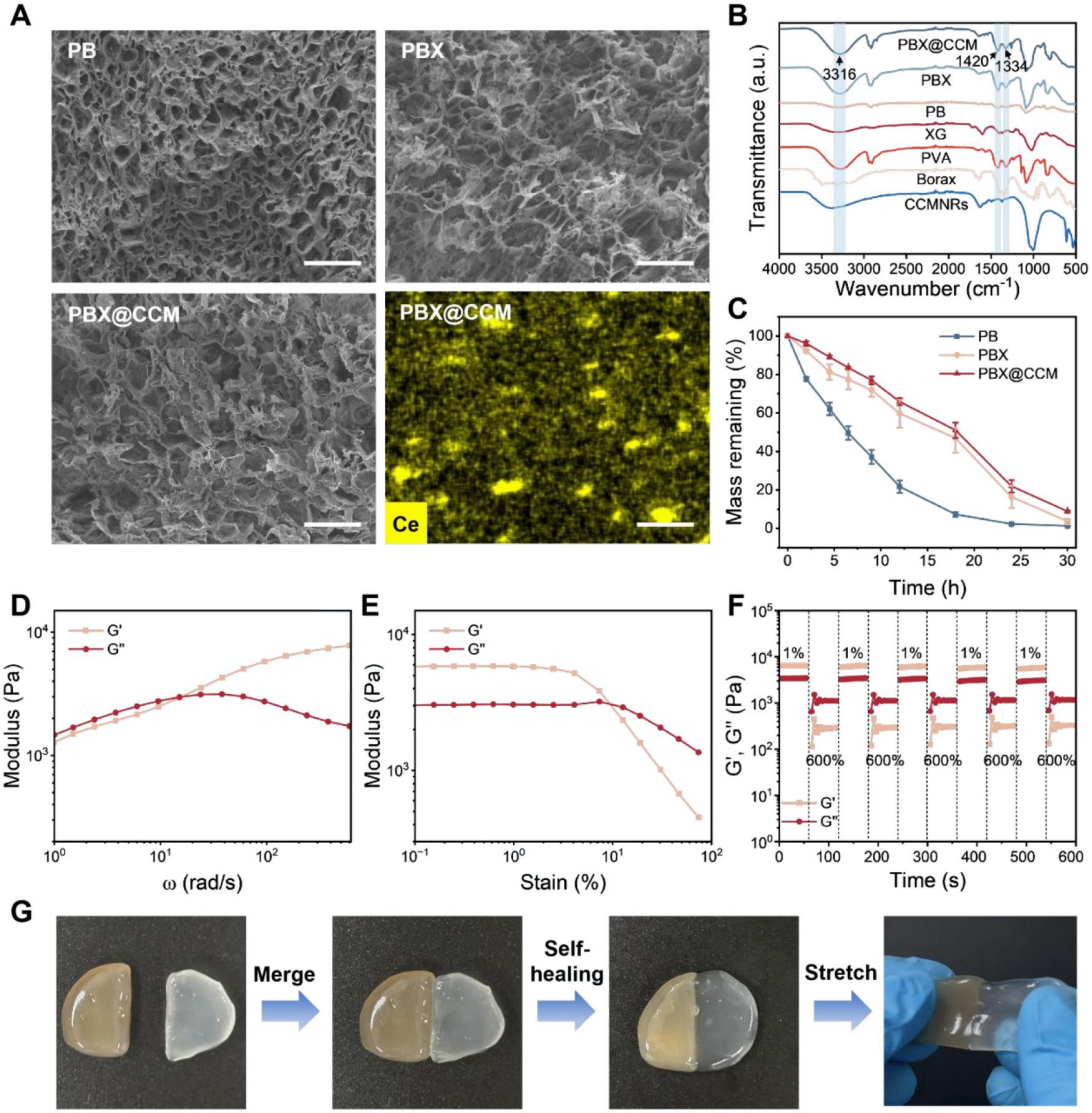
Preparation and characterization of PBX@CCM hydrogel. (**A**) Representative SEM images of PB, PBX and PBX@CCM hydrogels and EDS mapping of Ce in PBX@CCM hydrogel. Scale bar = 20 µm. (**B**) FT-IR spectra of PB, PBX and PBX@CCM hydrogels and the individual components. (**C**) Degradation curves of PB, PBX and PBX@CCM hydrogels in PBS at 37°C (*n* = 3). (**D**) Frequency sweep of PBX@CCM hydrogel over the range of 1–600 rad/s. (**E**) Strain sweep of PBX@CCM hydrogel over the range of 0.1–100%. (**F**) Rheological properties of PBX@CCM hydrogel over five cycles under alternating low (1%) and high (600%) strain. (**G**) Photographs demonstrating the macroscopic self-healing capability of PBX@CCM hydrogel.

The surface morphology of the hydrogel was analysed using SEM. As shown in Figure 4A, the PB hydrogel exhibited a porous structure with pore diameters of approximately 10–20 µm and relatively thick pore walls. After incorporation of XG, the pore walls became thinner, and an interconnected, crosslinked porous network was formed. Notably, the addition of CCMNRs did not markedly alter the pore size or overall network structure of the PBX hydrogel. The EDS mapping of the PBX@CCM hydrogel revealed a uniform distribution of cerium on the pore walls and within the pores, confirming successful incorporation and homogeneous dispersion of CCMNRs throughout the hydrogel network. The structure of PBX@CCM hydrogel was confirmed by FT-IR analysis (Figure 4B). A broad band at 3316 cm^−1^ was attributed to O–H stretching, reflecting hydrogen bonding between PVA and XG. Two additional characteristic peaks at 1420 and 1334 cm^−1^ were assigned to the asymmetric stretching of B–O–C, indicating the formation of borate ester linkages between borax, PVA and XG [37]. These results show that the PBX@CCM network is constructed through a combination of hydrogen bonds and borate ester bonds. The degradation profile further revealed that incorporation of XG slowed the degradation rate (Figure 4C), which can be attributed to the enhanced hydrogen bonding between PVA and XG. As shown in Supplementary Figure S10, MYR was gradually released as the hydrogel degraded, with complete release achieved within 32 h. This release behavior is consistent with the staged antioxidant strategy, in which MYR acts as a first responder during the early inflammatory phase, while the ceria nanozymes provide sustained ROS clearance thereafter. Dynamic rheological measurements were used to investigate the mechanical properties of PBX@CCM hydrogel. As expected, the PBX@CCM hydrogel displayed a typical hydrogel behavior, with the storage modulus (*G*′) higher than the loss modulus (*G*″) (Figure 4D). Strain sweep tests showed that PBX@CCM hydrogel maintained structural integrity up to about 10% strain (Figure 4E). When subjected to large strain, the internal network was temporarily disrupted, but the hydrogel rapidly self-recovered its original mechanical properties once the strain was removed (Figure 4F). Macroscopically, cut hydrogel pieces could rejoin and heal into an intact form without external assistance (Figure 4G), demonstrating the good self-healing capability of PBX@CCM, which is advantageous for adapting to wound motion and repeated mechanical disturbance *in vivo*.

### Biocompatibility of PBX@CCM hydrogel

**Figure 5:**
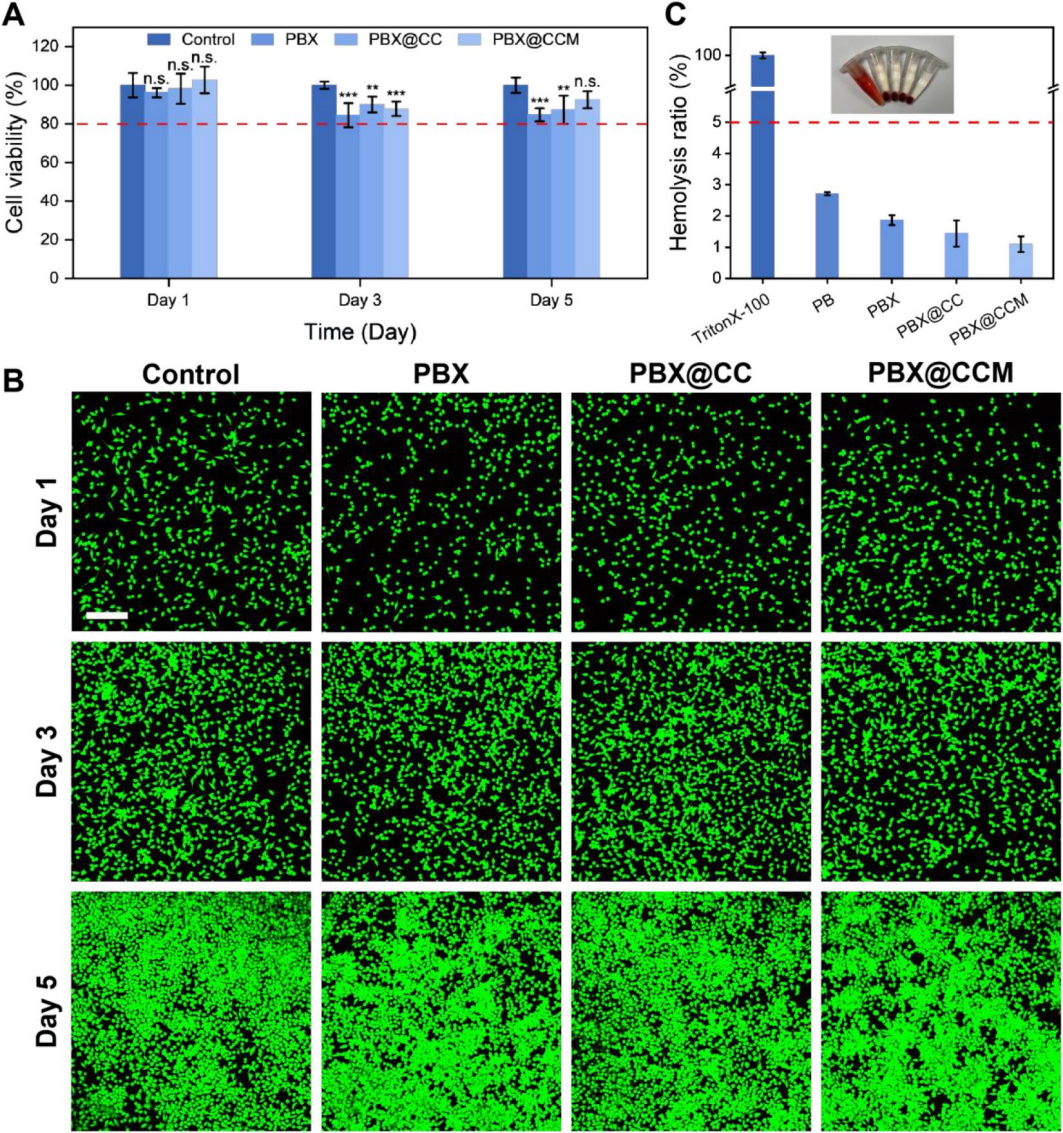
Biocompatibility of PBX@CCM hydrogel. (**A**) Viability of L929 cells after incubation with hydrogel extracts for 1, 3 and 5 days (*n* = 6). ** and *** indicate *P *< 0.01 and *P *< 0.001 compared with the control group, respectively. n.s. indicates non-significant. (**B**) Representative live/dead staining images of L929 cells after co-culture with hydrogel extracts for 1, 3 and 5 days. Scale bar = 100 µm. (**C**) Hemolysis ratio of rat RBCs treated with hydrogel extracts (*n* = 5).

To verify that the hydrogel is suitable for wound care applications, both cytotoxicity and hemocompatibility were systematically evaluated. First, the cytotoxicity of the hydrogel was assessed using L929 fibroblasts cultured with the corresponding hydrogel extracts. Cell viability was quantified using CCK-8 assay. As shown in Figure 5A, because borax exhibits mild cytotoxicity that increases over time [38], cell viability in the PBX, PBX@CC and PBX@CCM groups all decreased by day 3 of the co-culture; on day 5, the inhibitory effect on cells in the PBX group persisted, while the PBX@CC group showed a slight recovery in cell viability due to the antioxidant effects of cerium, though the difference from the control group remained significant. In contrast, in the PBX@CCM group, MYR and ceria provided complementary antioxidant and cytoprotective effects [39], effectively alleviating the cytotoxic stress from the hydrogel matrix and improving cell viability to a level showing no significant difference from the control group. Overall, cell viability in all groups remained above 80% on days 1, 3 and 5, confirming that the three hydrogels exhibit acceptable biocompatibility and that the loading of CCMNRs significantly enhances the long-term cytocompatibility of the hydrogels. These results were further supported by live/dead staining. Fluorescence images (Figure 5B) revealed that L929 cells exposed to PBX, PBX@CC and PBX@CCM hydrogel extracts for 1, 3 and 5 days predominantly exhibited green fluorescence (live cells), while red fluorescence (dead cells) was rarely observed. The cell morphology also remained normal, with no obvious signs of shrinkage or detachment. Together, these results demonstrate that the hydrogels possess good cytocompatibility. Hemocompatibility was evaluated by a hemolysis assay using rat RBCs. Blood samples treated with physiological saline and with extracts of PB, PBX, PBX@CC and PBX@CCM remained clear, whereas those treated with Triton X-100 turned red due to hemolysis (Figure 5C). Quantitative analysis revealed that the hemolysis ratios for all hydrogel groups were below 5%, which is generally considered acceptable for blood-contacting materials. These findings indicate that PB, PBX, PBX@CC and PBX@CCM hydrogels exhibit low hemolytic risk and are suitable for further *in vivo* wound applications.

### Antioxidant and oxygen generation ability of PBX@CCM hydrogel

**Figure 6:**
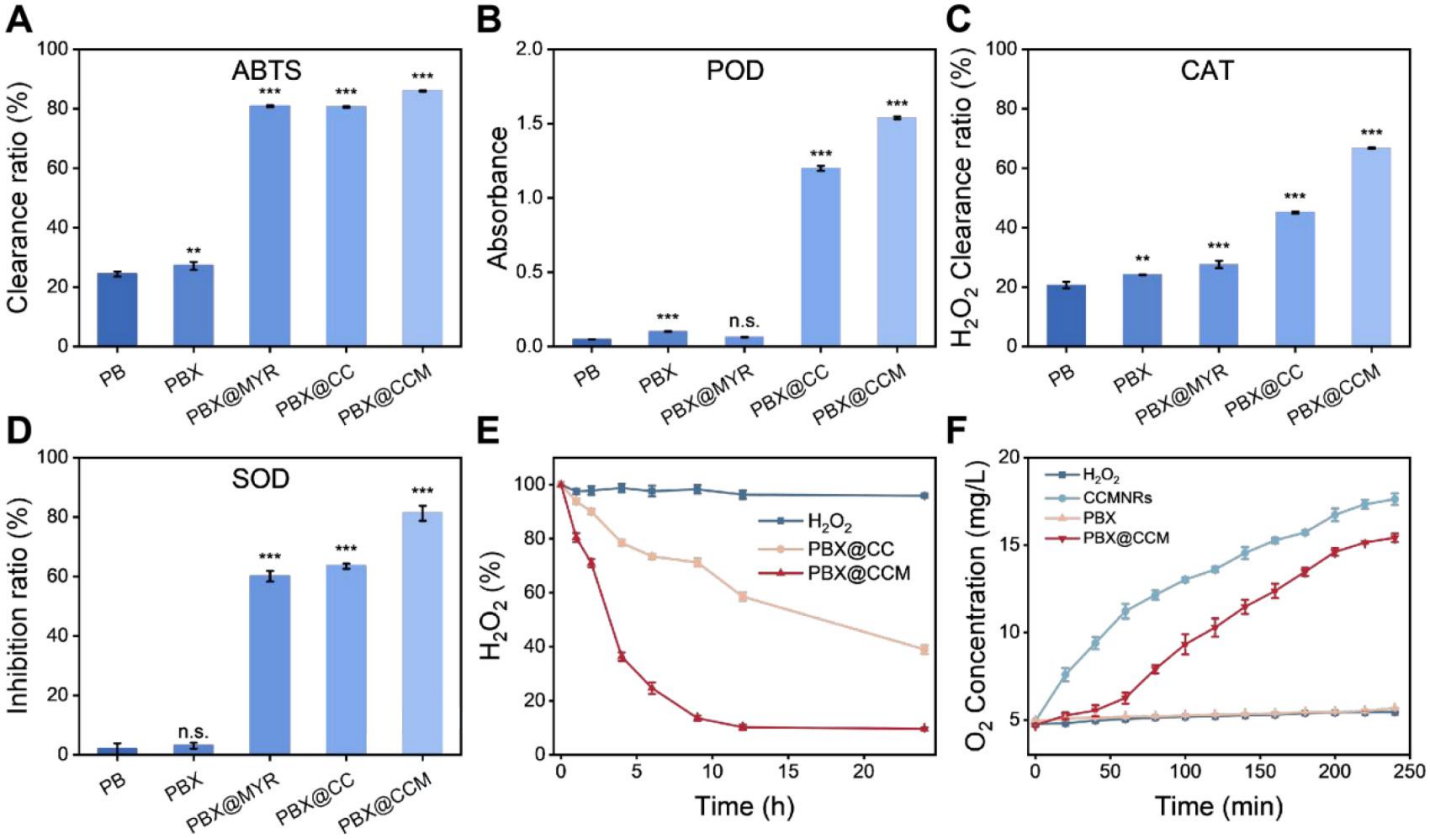
Radical scavenging capacity and multi-enzyme mimetic activities of different hydrogels. (**A**) ABTS radical scavenging capacity, (**B**) POD, (**C**) CAT and (**D**) SOD mimetic enzyme activities of PB, PBX, PBX@MYR, PBX@CC and PBX@CCM hydrogels (*n* = 3). ** and *** indicate *P *< 0.01 and *P *< 0.001 compared with the PB group, respectively. n.s. indicates non-significant. (**E**) H_2_O_2_ scavenging activity of hydrogel samples in solution, measured using titanyl sulfate as the H_2_O_2_-specific probe (*n* = 3). (**F**) Oxygen production profiles over time for CCMNRs and PBX@CCM in H_2_O_2_ solutions (*n* = 3).

Hyperglycemia leads to excessive ROS generation and impaired oxygen supply, both of which severely disrupt the healing process in diabetic wounds [40]. Given the strong, auto-regenerative antioxidant performance of CCMNRs, we subsequently incorporated these nanozymes into PBX hydrogel. To evaluate whether the loaded CCMNRs maintained sustained antioxidant capacity, the hydrogels were treated with H_2_O_2_. The results showed that PBX@CC and PBX@CCM hydrogels changed from nearly white and dark brown to yellow and brown-yellow, respectively, owing to the oxidation of Ce^3+^ to Ce^4+^, and gradually returned to their initial colors after standing for 1 day indicating spontaneous Ce^3+^ regeneration (Supplementary Figure S11). Upon re-exposure to H_2_O_2_, the color changed again, confirming that the Ce^3+^/Ce^4+^ redox cycling of CCMNRs loaded into the hydrogels remained functional, thereby ensuring sustained ROS scavenging capability. To systematically assess the hydrogels’ radical scavenging ability and multi-enzyme mimetic activity, we measured the ABTS radical scavenging capacity and the enzyme activities of POD, CAT and SOD for each sample group (Figure 6A–D). The results indicated that the ABTS radical scavenging rates and enzyme activities (POD, CAT and SOD) of PB and PBX hydrogels were relatively low, suggesting that the PVA/XG/borax three-dimensional network did not confer antioxidant properties. In contrast, the PBX@MYR hydrogel loaded with MYR exhibited significantly enhanced ABTS radical scavenging capacity (80.2%) and notable superoxide anion scavenging ability in the SOD assay (60.1%), attributable to the direct chemical reduction of O_2_·^−^ by the phenolic hydroxyl groups in MYR [41, 42]. However, its CAT and POD enzyme activities remained low, confirming that MYR exerts its antioxidant effects mainly through direct radical scavenging rather than enzyme-mimicking catalysis. In contrast, the CCNRs-loaded PBX@CC hydrogel demonstrated ABTS radical scavenging efficiency comparable to that of PBX@MYR hydrogel (80.6%) and exhibited robust activity of POD, CAT and SOD enzymes, indicating that ceria primarily exerts its antioxidant effects through multi-enzyme mimetic activity. Finally, the PBX@CCM hydrogel, which combines MYR and CCNRs, exhibited optimal comprehensive antioxidant performance: its ABTS radical scavenging ratio increased to 85.9%, and the enzyme activities of POD, CAT and SOD reached the highest values among all groups, significantly outperforming PB, PBX, PBX@MYR and PBX@CC. Notably, while the ABTS scavenging rates of PBX@MYR and PBX@CC were comparable, their antioxidant mechanisms are fundamentally distinct: MYR contributes stoichiometric radical scavenging, whereas ceria provides catalytic, regenerable ROS elimination. This mechanistic complementarity, rather than simple additive scavenging, underlies the enhanced overall performance of PBX@CCM. Thus, we conclude that MYR primarily relies on rapid direct radical scavenging, while CCNRs demonstrate their core advantage in long-lasting antioxidant effects through multi-enzyme catalysis. The combination of both components in PBX@CCM yields complementary antioxidant performance.

The H_2_O_2_ scavenging capacity of the hydrogel was further evaluated using an ultraviolet–visible calibration curve based on the absorbance of the titanium sulfate and hydrogen peroxide at 415 nm (Supplementary Figure S12). After incubation with PBX@CC hydrogel, approximately 42% of H_2_O_2_ was cleared within 12 h, and nearly 60% within 24 h. PBX@CCM hydrogel exhibited much faster kinetics, eliminating about 90% of H_2_O_2_ within 12 h (Figure 6E). This is consistent with the catalytic cycle of ceria, where Ce^4+^ decomposes H_2_O_2_ to generate Ce^3+^ and O_2_, enabling ceria to act as an oxygen reservoir and supplier [43]. The faster H_2_O_2_ clearance by PBX@CCM compared to PBX@CC is not due to the direct decomposition of H_2_O_2_ by MYR, as free MYR itself has almost no ability to directly catalyse H_2_O_2_ decomposition (Supplementary Figure S13), but rather is achieved through an indirect mechanism in which MYR promotes the regeneration of Ce^3+^ on the surface of ceria, effectively maintaining the reversible Ce^3+^/Ce^4+^ redox cycle [44, 45]. This sustains the enzyme-like catalytic activity of ceria, thereby efficiently accelerating H_2_O_2_ decomposition and improving antioxidant efficacy in the composite hydrogel. The dissolved oxygen content in the reaction system increased progressively in the presence of CCMNRs and PBX@CCM hydrogel (Figure 6F), indicating that PBX@CCM can continuously generate O_2_ under high-ROS conditions.

In addition to oxidative stress, AGEs formed via Maillard reactions between proteins and excess glucose are another important factor contributing to delayed wound healing in diabetes. Both ceria and MYR have been reported to exhibit AGEs-inhibitory effects [46, 47]. The fluorescence intensity of AGEs formed in bovine serum albumin–glucose systems treated with different hydrogels (Supplementary Figure S14) showed that both PBX@CC and PBX@CCM reduced AGEs formation compared to the control, with PBX@CCM giving the strongest inhibitory effect. The inhibition of AGEs formation by PBX@CCM hydrogel can be attributed to the complementary actions of its two functional components. MYR can directly scavenge free radicals and capture reactive carbonyl intermediates, thereby blocking early glycation reactions at their source [48]. Meanwhile, ceria nanozymes possess a reversible Ce^3+^/Ce^4+^ redox pair and multiple enzymatic activities, enabling them to efficiently scavenge ROS and thereby interrupt the oxidative chain reaction of glycation [49]. Together, these two components work through different mechanisms to jointly inhibit the formation of AGEs. Overall, these data indicate that PBX@CCM hydrogel not only provides robust ROS scavenging and O_2_ generation but also helps to limit AGEs accumulation, thereby alleviating oxidative stress and hypoxia in the diabetic wound microenvironment and offering a more protective milieu for cell survival and tissue repair.

### Intracellular ROS scavenging and immunomodulatory capacity of PBX@CCM hydrogel

**Figure 7:**
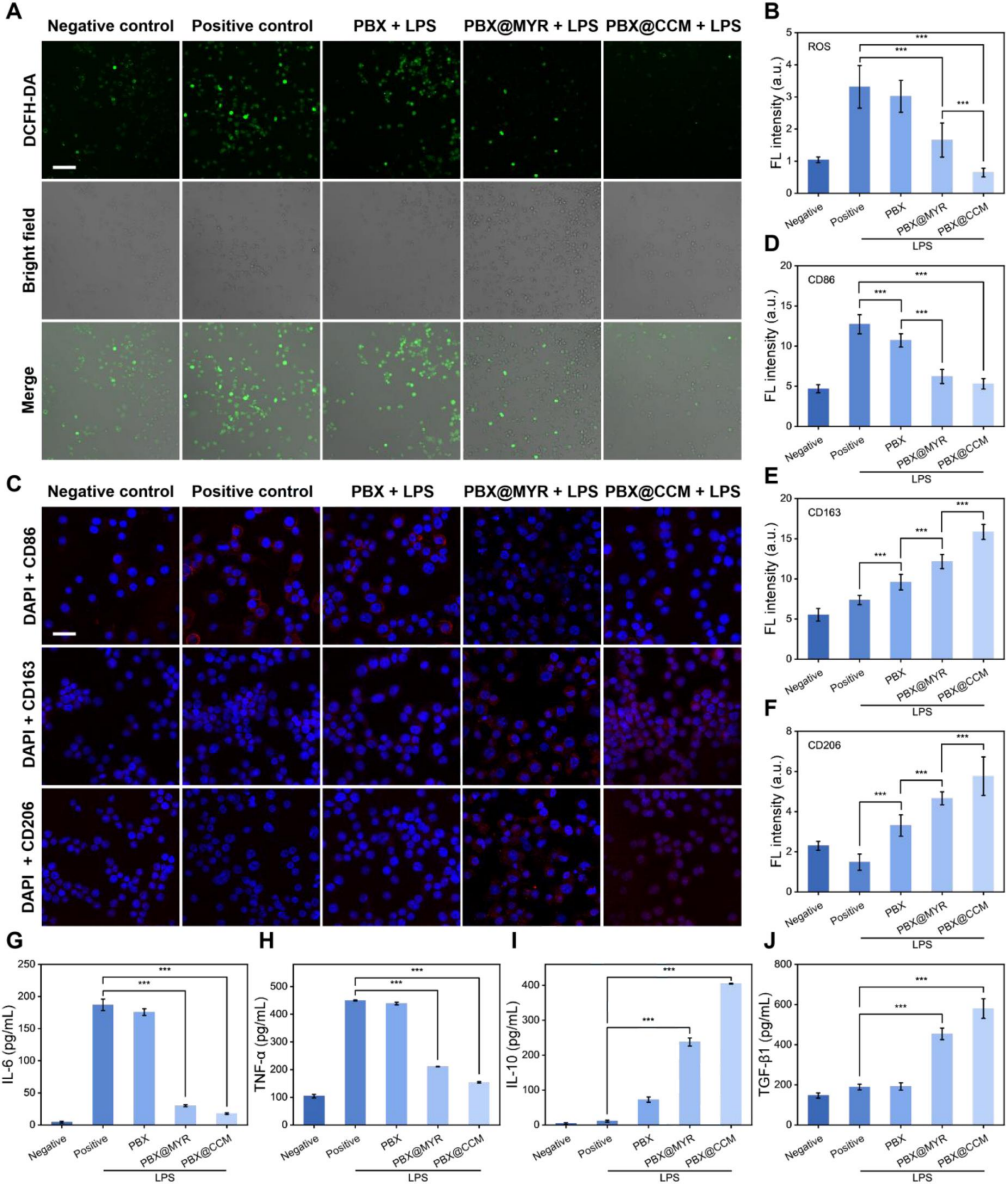
Intracellular ROS scavenging and immunomodulatory abilities of different hydrogels under LPS stimulation. (**A**) Representative confocal fluorescence images showing ROS expression in RAW 264.7 cells treated with different hydrogel extracts, using DCFH-DA probes to assess ROS levels. Scale bar = 100 µm. (**B**) Quantitative results of ROS fluorescence intensity. (**C**) Representative confocal fluorescence images of CD86, CD163 and CD206 expression in RAW 264.7 cells treated with different hydrogel extracts, evaluating the anti-inflammatory effects. Scale bar = 20 µm. (**D–F**) Quantitative results of CD86, CD163 and CD206 fluorescence intensity (*n* = 3). (**G–J**) ELISA quantification of inflammation-related cytokines IL-6, TNF-α, IL-10 and TGF-β1 in the culture supernatants of RAW 264.7 cells (*n* = 3).

Excess ROS accumulate within the diabetic wound environment, resulting in a chronic state of oxidative stress [50]. The above *in vitro* ROS scavenging experiments demonstrated that the PBX@CCM hydrogel exhibited excellent antioxidant capacity. To further validate the hydrogel’s ROS clearance capability at the cellular level, we established LPS- and H_2_O_2_-induced macrophage oxidative stress models to generate intracellular ROS. As shown in Figure 7A, clear intracellular fluorescence was observed in the LPS-stimulated group (positive control), indicating a significant increase in intracellular ROS levels. Treatment with PBX showed no significant difference compared with the LPS group, suggesting that PBX alone did not effectively reduce ROS levels. In contrast, the fluorescence signal in the PBX@MYR treatment group was significantly attenuated, while the ROS levels in the PBX@CCM treatment group decreased to levels comparable to those in the negative control group (Figure 7B), demonstrating the strongest intracellular ROS clearance among all treatment groups. To more accurately simulate oxidative damage at the wound site, we further established an H_2_O_2_-induced cellular oxidative stress model. As shown in Supplementary Figure S15A, PBX@CCM and PBX@MYR hydrogels significantly reduced intracellular ROS levels under H_2_O_2_-induced oxidative stress. Their antioxidant protective effects were significantly superior to those of the PBX and H_2_O_2_ groups, with the PBX@CCM group exhibiting the strongest intracellular ROS scavenging capacity (Supplementary Figure S15B). These findings are consistent with the conclusions from the LPS-induced oxidative stress model, confirming that the PBX@CCM hydrogel not only inhibits indirect ROS generation mediated by inflammatory pathways but also efficiently scavenges exogenous H_2_O_2_-derived ROS, demonstrating robust antioxidant capacity.

Macrophages are the major immune cells in skin wounds, typically polarizing into pro-inflammatory M1 or pro-reparative M2 subtypes under microenvironmental cues, playing an important role in wound repair [51]. To investigate the immunomodulatory and anti-inflammatory effects of PBX@CCM hydrogel, we used LPS to mimic the inflammatory microenvironment of diabetic wounds. We then examined the expression of marker proteins associated with M1 (CD86) and M2 (CD163 and CD206) macrophage polarization using immunofluorescence (Figure 7C). The results demonstrated that LPS stimulation significantly upregulated the expression of the M1 macrophage marker CD86 and suppressed the expression of M2 markers CD163 and CD206. The PBX treatment group showed no significant effect on macrophage polarization. The PBX@MYR treatment group partially inhibited CD86 expression while upregulating CD163 and CD206. The PBX@CCM treatment group markedly reduced CD86 fluorescence intensity and substantially increased the expression levels of CD163 and CD206, exhibiting the strongest M2 polarization-inducing capability. Semi-quantitative analysis further confirmed these trends (Figure 7D–F), demonstrating that PBX@CCM possesses the most pronounced anti-inflammatory and immunomodulatory effects among all groups. To quantitatively evaluate the immunomodulatory effects of the hydrogel, the expression levels of key inflammatory cytokines in LPS-stimulated macrophages were measured by ELISA (Figure 7G–J). Compared to the positive control group, the secretion of pro-inflammatory cytokines IL-6 and TNF-α was significantly inhibited in the PBX@MYR and PBX@CCM hydrogel treatment groups, with the most pronounced inhibition observed in the PBX@CCM group. Concurrently, the levels of anti-inflammatory cytokines IL-10 and TGF-β1 exhibited a stepwise and significant upregulation, with the highest levels recorded in the PBX@CCM group. These results provide direct quantitative evidence that the PBX@CCM hydrogel effectively promotes macrophage polarization from the pro-inflammatory M1 phenotype to the anti-inflammatory M2 phenotype, thereby alleviating local inflammation. These findings suggest that PBX@CCM hydrogel can effectively reduce cellular oxidative stress and promote the polarization of macrophages from the M1 to M2 phenotype. This shift, coupled with reduced inflammatory responses, offers a promising strategy to mitigate inflammation in diabetic wounds.

### *In vivo* assessment of diabetic wound healing

**Figure 8:**
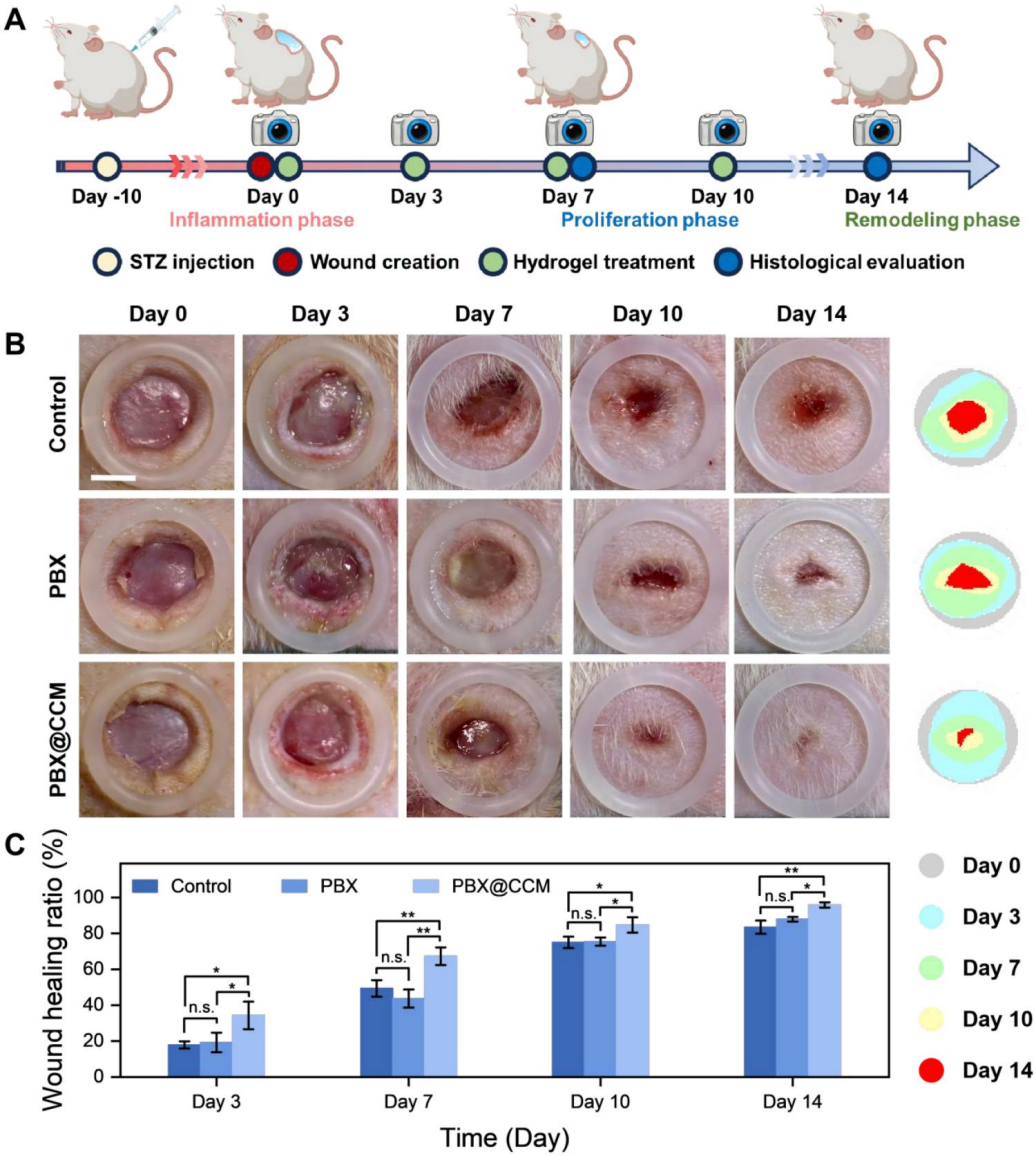
*In vivo* evaluation of diabetic wound-healing efficacy using PBX@CCM hydrogel dressing. (**A**) Schematic representation of the diabetic wound animal model and treatment regimen. (**B**) Photographs of wounds treated with different materials at days 0, 3, 7, 10 and 14, showing the wound bed closure over the 14-day period for all three groups. Scale bar = 5 mm. (**C**) Statistical analysis of wound healing ratio over the treatment period (*n* = 8).

After confirming the good biocompatibility, ROS-scavenging capacity and anti-inflammatory properties of the hydrogel, its therapeutic efficacy in diabetic wound healing was further evaluated *in vivo* using SD rats (Figure 8A). Diabetes was induced by intraperitoneal injection of STZ for three consecutive days, and non-fasting blood glucose levels ≥16.7 mM were used as the diagnostic criterion. Persistent hyperglycemia was monitored to confirm successful model establishment. Full-thickness circular wounds (8 mm in diameter) were then created on the dorsal skin of diabetic rats, which were randomly assigned to three groups: saline (control), PBX hydrogel and PBX@CCM hydrogel. Over the 14-day observation period, the PBX@CCM hydrogel-treated group showed markedly accelerated wound closure compared to the control and PBX hydrogel groups (Figure 8B). The PBX group showed only limited improvement relative to the control group, indicating that the base hydrogel matrix alone contributed mainly to moisture retention rather than active biofunction regulation. In contrast, PBX@CCM treatment resulted in obvious shrinkage of the wound area and faster formation of new tissue. Quantitative analysis of the relative wound healing ratio further confirmed these findings (Figure 8C). On day 7, the PBX@CCM hydrogel group achieved 67.3% closure, significantly higher than the control (49.4%) and PBX hydrogel (43.7%) groups. By day 14, wounds treated with PBX@CCM reached approximately 95.9% wound closure with extensive re-epithelialized, compared with 83.5% for the control group and 87.9% for the PBX hydrogel group. These data indicate that PBX@CCM hydrogel markedly accelerates the healing of diabetic wounds, which can be attributed to its combined rapid-and-sustained antioxidant activity, oxygen generation and immunomodulatory effects. It should be noted that the absence of a PBX@CC group in the *in vivo* animal experiments presents a limitation. Although *in vitro* data have clearly demonstrated the complementary effect between ceria nanozymes and MYR, *in vivo* results alone cannot distinguish whether the therapeutic advantage of PBX@CCM over PBX is attributable to ceria, MYR or their combination. Further studies incorporating additional control groups in animal experiments would help to fully delineate the individual contributions of each component *in vivo*.

**Figure 9:**
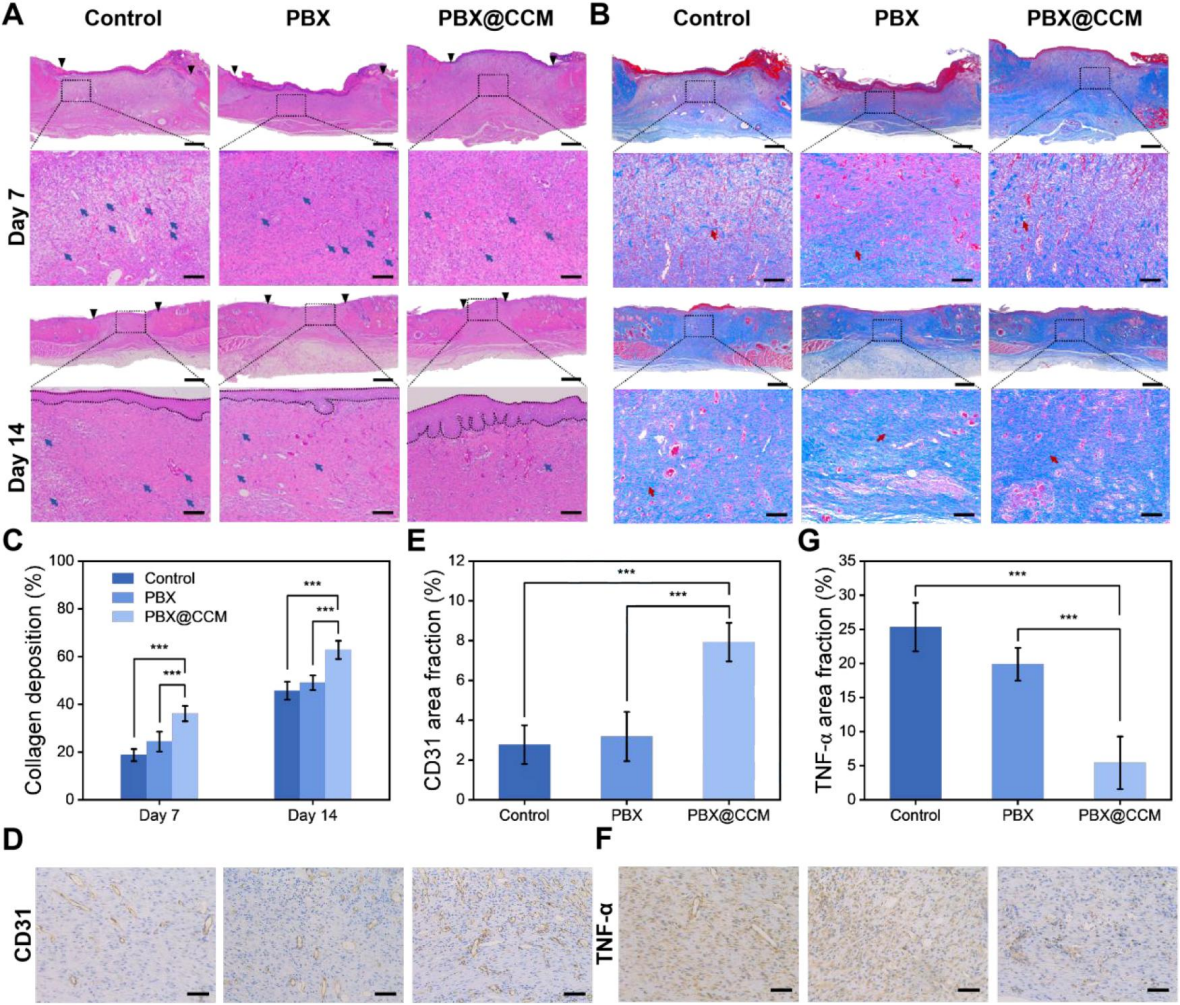
Histological and immunohistochemical analysis of wound healing. (**A**) H&E-stained wound sections. The region between the two black triangles indicates the wound area. Blue arrows denote inflammatory cells. Scale bar = 500 µm (low magnification), 50 µm (high magnification). (**B**) Masson’s trichrome-stained wound sections. Red arrows indicate collagen fibers. Scale bar = 500 µm (low magnification), 50 µm (high magnification). (**C**) Quantitative analysis of collagen deposition based on Masson’s trichrome staining (*n* = 5). (**D**) Immunohistochemical staining of CD31 in wound tissues at day 14 post-injury. Scale bar = 50 µm. (**E**) Quantitative analysis of CD31-positive area (*n* = 5). (**F**) Immunohistochemical staining of TNF-α in wound tissues at day 14 post-injury. Scale bar = 50 µm. (**G**) Quantitative analysis of TNF-α-positive area (*n* = 5).

Histological analysis and immunohistochemical staining were performed to further evaluate the wound-healing efficacy of the PBX@CCM hydrogel. H&E staining (Figure 9A) showed that at days 7 and 14, the wound area in the PBX@CCM hydrogel group was smaller compared to the control and PBX groups, with visibly fewer inflammatory cells in the wound region. By day 14, all groups exhibited re-epithelialization. However, the PBX@CCM hydrogel group exhibited a markedly thicker epidermal layer, indicating more advanced tissue regeneration. Masson’s trichrome staining was used to assess collagen deposition (Figure 9B). Collagen fibers in the control and PBX groups appeared relatively loose and sparsely distributed, whereas the PBX@CCM hydrogel group exhibited denser and more organized collagen alignment. Quantitative analysis (Figure 9C) confirmed that collagen deposition in the PBX@CCM hydrogel group was significantly higher than in the control and PBX hydrogel groups at both days 7 and 14, suggesting that PBX@CCM hydrogel promotes extracellular matrix remodeling. Neovascularization was evaluated by CD31 immunostaining (Figure 9D). The PBX@CCM hydrogel group showed stronger CD31-positive staining and more abundant microvasculature compared with the other groups. Quantitative results (Figure 9E) indicated that the relative CD31-positive area in the PBX@CCM group was approximately 2.8-fold higher than in the control group and 2.5-fold higher than in the PBX group, demonstrating enhanced angiogenic activity. TNF-α, a key pro-inflammatory cytokine, was used as a marker to assess the local inflammatory response. Immunohistochemical staining and semi-quantitative analysis of TNF-α (Figure 9F and G) showed that the expression was markedly reduced in the PBX@CCM hydrogel group compared with the control and PBX groups, indicating attenuation of the inflammatory microenvironment. Taken together, these histological and immunohistochemical results support that PBX@CCM hydrogel can simultaneously reduce inflammation, promote collagen deposition and enhance angiogenesis, thereby effectively accelerating diabetic wound healing and presenting itself as a promising candidate for advanced chronic wound dressings.

## Conclusion

In this study, a multifunctional PBX@CCM hydrogel was developed by incorporating MYR and ceria nanozymes (CCMNRs) into a dynamic PBX hydrogel matrix, yielding a complementary dual-action antioxidant and immunomodulatory wound dressing. MYR rapidly scavenges ROS, effectively neutralizing oxidative bursts in the early stages of wound healing, while ceria nanozymes provide sustained ROS clearance through their auto-regenerative redox cycling between Ce^3+^ and Ce^4+^. This combination enhances the hydrogel’s antioxidant capacity, ensuring both immediate protection and long-term defense against oxidative stress. The hydrogel exhibits multi-enzyme-mimicking activities, including POD-, SOD- and CAT-like functions, further strengthening its therapeutic potential. The PBX@CCM hydrogel demonstrated good biocompatibility, effectively reduced cellular oxidative stress and promoted macrophage polarization from the M1 pro-inflammatory phenotype to the M2 anti-inflammatory phenotype. *In vivo* experiments using a diabetic rat model revealed accelerated wound closure and improved tissue regeneration, emphasizing the potential of this hydrogel as an effective therapeutic strategy for chronic diabetic wounds. Overall, this study presents a promising approach for treating diabetic wounds, combining rapid-and-sustained antioxidant effects with immune modulation to enhance wound healing outcomes.

## Supplementary Material

rbag105_Supplementary_Data

## Data Availability

The data that support the findings of this study are available from the corresponding author upon reasonable request.
