# Supplementary material for: Complementary antioxidant strategy for diabetic wound healing: myricetin and ceria nanozymes for enhanced reactive oxygen species management
Source: Regen Biomater. 2026 Jun 2;13:rbag105. doi: 10.1093/rb/rbag105 (PMC13303295; doi:10.1093/rb/rbag105)
Supplement: rbag105_Supplementary_Data [file rbag105_supplementary_data.docx]

**Supporting Information**

**Complementary Antioxidant Strategy for Diabetic Wound Healing: Myricetin and Ceria Nanozymes for Enhanced Reactive Oxygen Species Management**

*Xiaoxiao Liao^1,2,3^,* *Lei Song^2,3^, Shancan Wang^2,3^, Kai Cheng^2,3^, Ying Xiao^2,3^, Donghong Li^2,3^, Shuyan Chen^2,3^, Yijing Liu^2,3^, Yinyu Zhao^2,3^, Xiaolin Zhang^1^, Yuechuan Shen^4*^, Menglan He^1*^, Rong Wang^2,3*^*

1, Marine Science and Technology College, Zhejiang Ocean University, Zhoushan, 316022, P. R. China.

2, Laboratory of Advanced Theranostic Materials and Technology, Ningbo Institute of Materials Technology and Engineering, Chinese Academy of Sciences, Ningbo 315201, P. R. China.

3, Zhejiang International Scientific and Technological Cooperative Base of Biomedical Materials and Technology, Ningbo Cixi Institute of Biomedical Engineering, Ningbo 315300, P. R. China.

4, Department of Emergency, Zhoushan Hospital of Zhejiang Province, Zhoushan, 316021, P. R. China.

Corresponding authors:

Y. Shen: shenyc0206@126.com

M. He: hemenglan@zjou.edu.cn

R. Wang: [rong.wang@nimte.ac.cn](mailto:rong.wang@nimte.ac.cn)

**Table S**1. Antibodies used in immunostaining

| **Antibody** | **Company** | **Category number** | **Application** | **Dilution** |
| --- | --- | --- | --- | --- |
| Anti-CD86 Antibody | Proteintech | 83213-1-RR | Immunofluorescence | 1:200 |
| Anti-CD163 Antibody | Abcam | ab316218 | Immunofluorescence | 1:400 |
| Anti-CD206 Antibody | Solarbio | K011692M | Immunofluorescence | 1:400 |
| Anti-CD31 Antibody | Servicebio | GB11063-2 | Immunohistochemistry | 1:600 |
| Anti-TNF-α Antibody | Servicebio | GB115701 | Immunohistochemistry | 1:500 |
| 568-conjugated Anti-rabbit IgG | Proteintech | SA00013-4 | Immunofluorescence | 1:200 |
| 568-conjugated Anti-mouse IgG | Solarbio | K1031G-AF568 | Immunofluorescence | 1:200 |
| HRP-Anti-rabbit IgG | Servicebio | GB23303 | Immunohistochemistry | 1:200 |

**
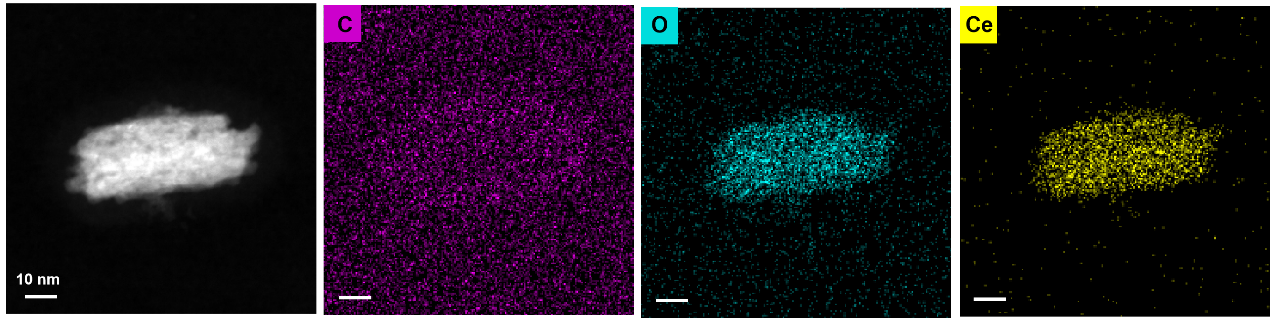
**

**Figure S1.** EDS elemental mapping of CCMNRs.

**
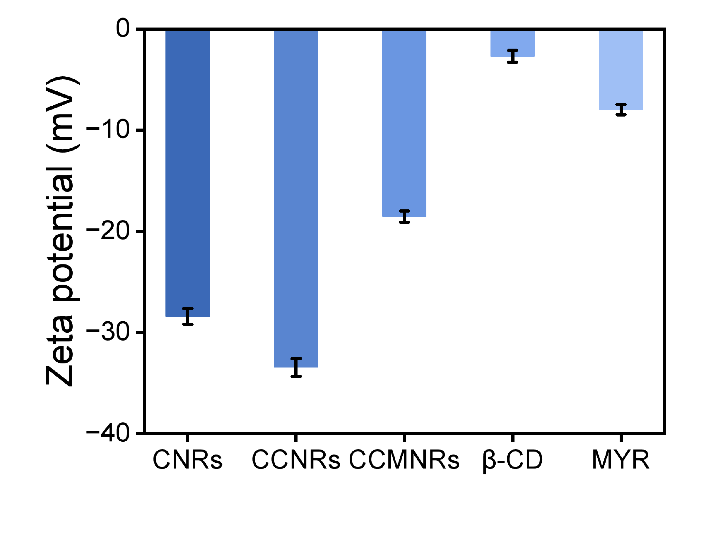
**

**Figure S2.** Zeta potentials of CNRs, CCNRs, CCMNRs, β-CD, and MYR in ultrapure water (n = 3).

**
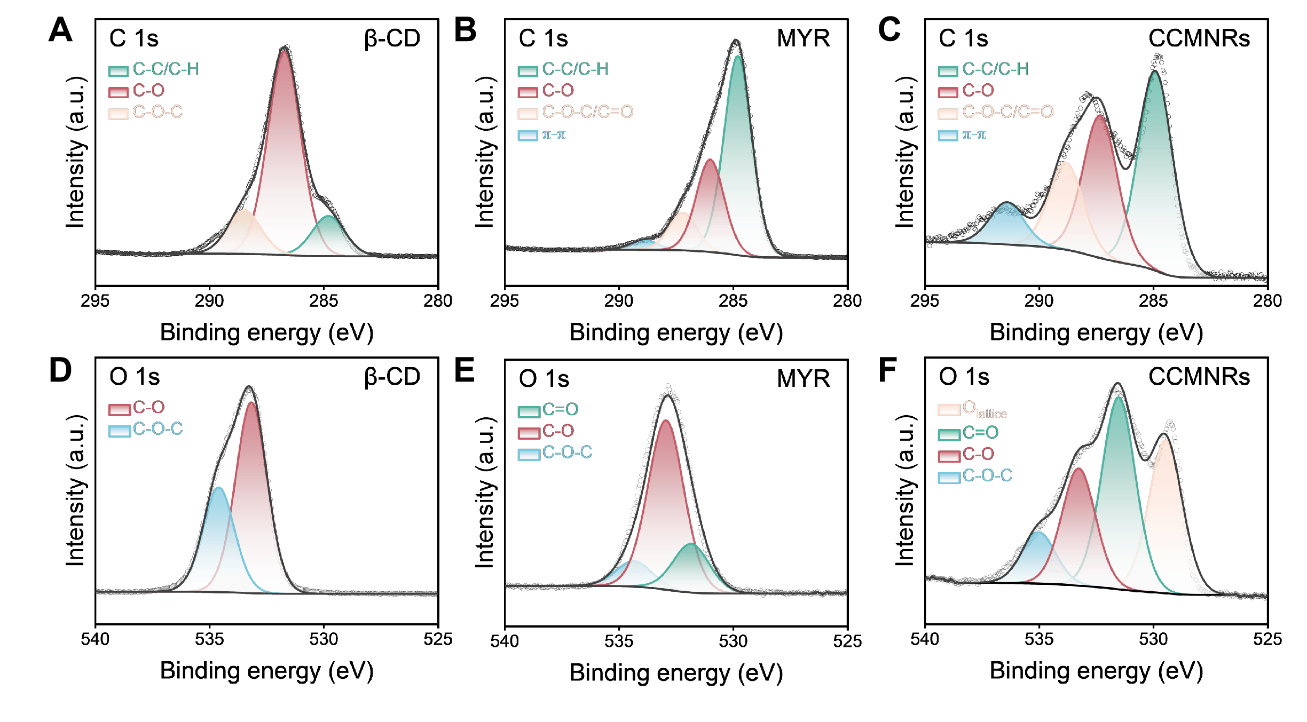
Figure S3.** XPS core-level spectra of (A-C) C 1s, and (D-F) O 1s for (A, D) β-CD, (B, E) MYR, and (C, F) CCMNRs.

**
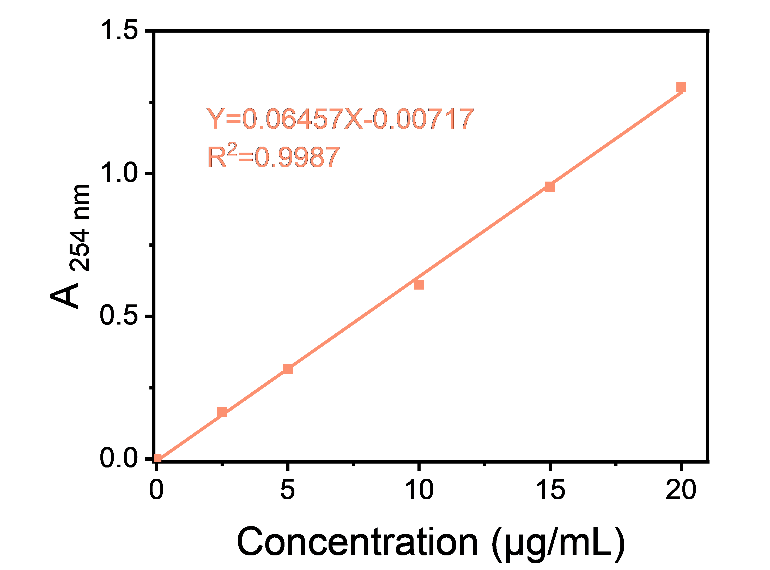
**

**Figure S4.** Standard calibration curve of MYR determined by ultraviolet-visible spectroscopy.

**
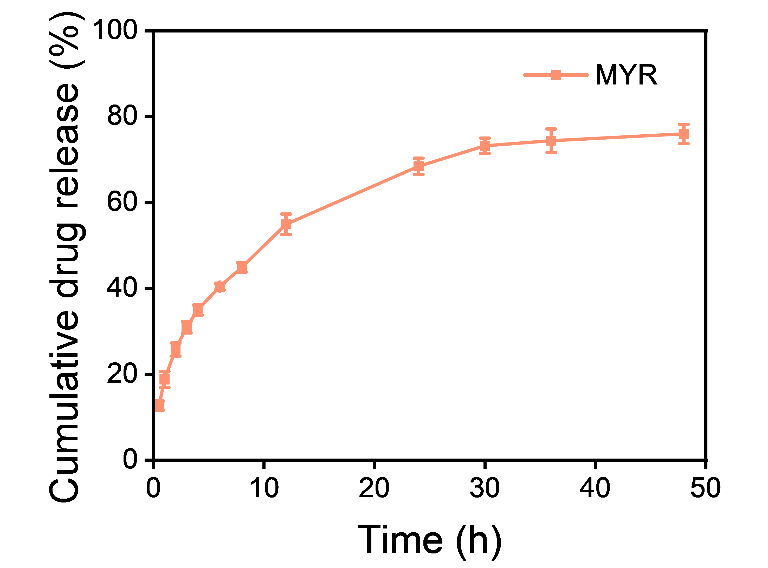
**

**Figure S5.** *In vitro* release profile of MYR from CCMNRs in PBS (10 mM, pH 7.4) at 37°C (n = 3).


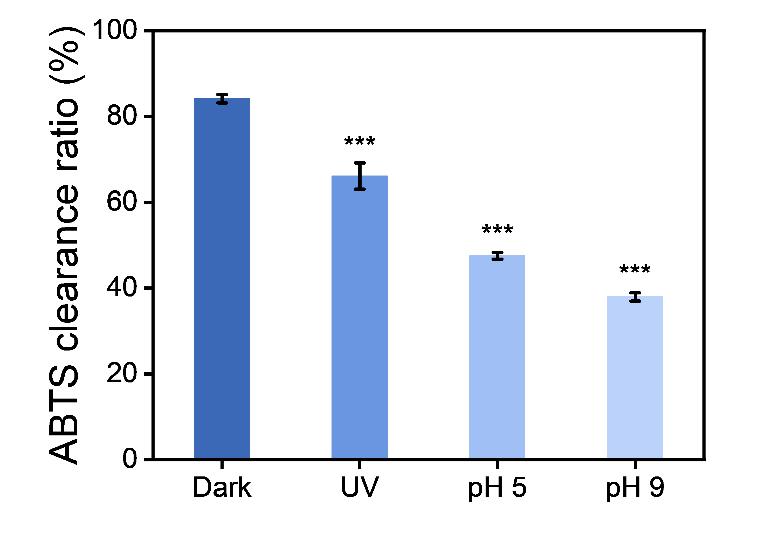


**Figure S6.** Stability of MYR under 6-h ultraviolet irradiation and different pH conditions (n = 3). *** indicates *p* < 0.001 compared with the dark group.

**
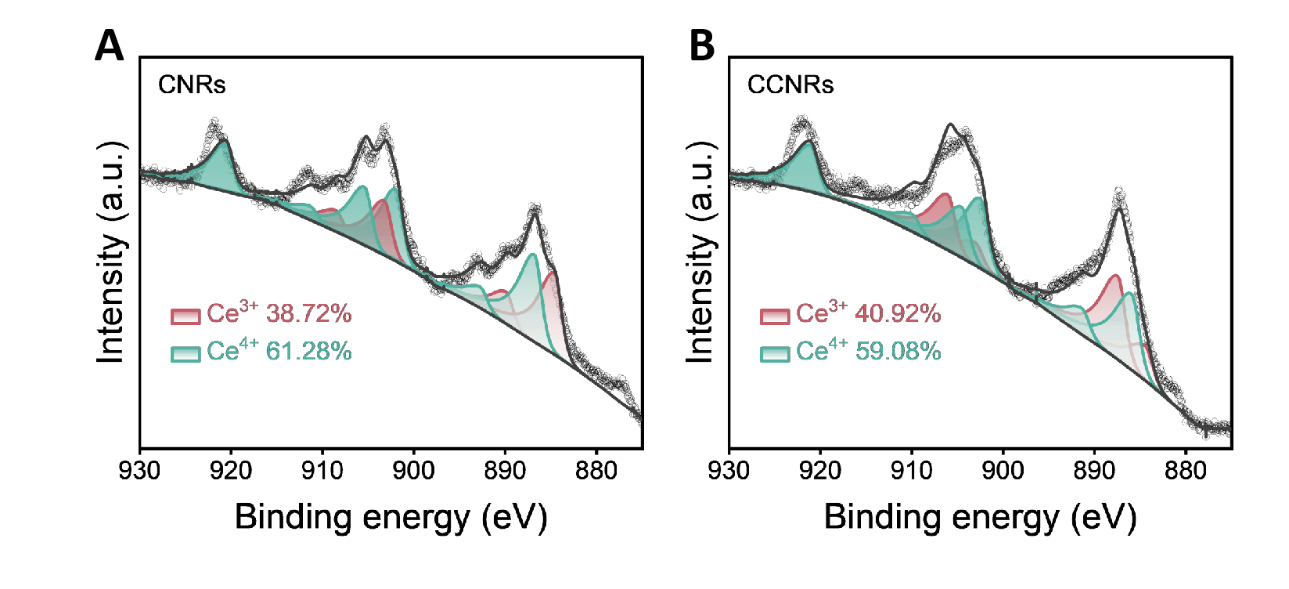
**

**Figure S7.** XPS Ce 3d core-level spectra for (A) CNRs and (B) CCNRs.


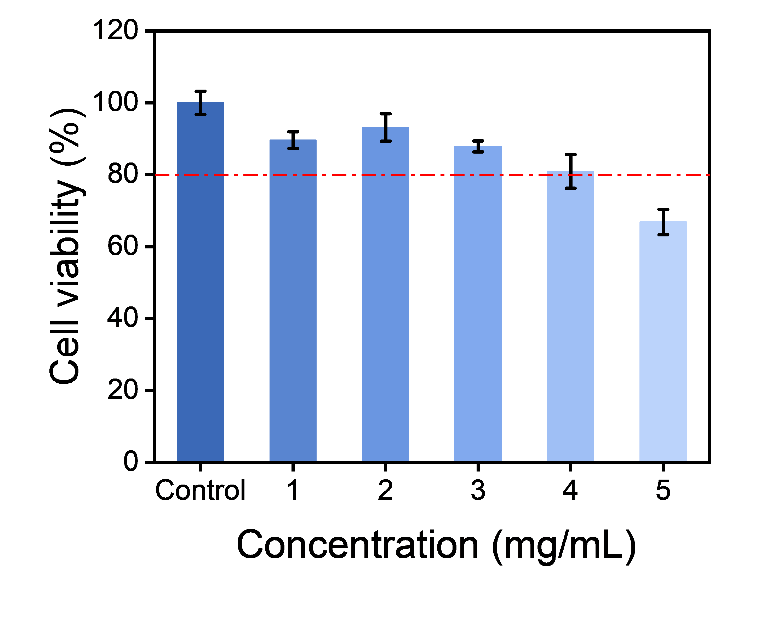


**Figure S8.** Viability of L929 cells after 24 h incubation with CCMNRs at different concentrations (n = 6).


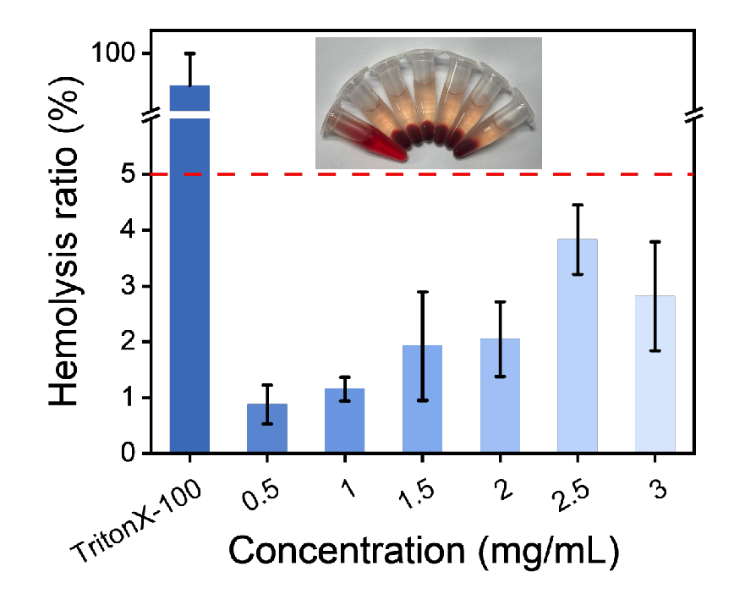


**Figure S9.** Hemolysis ratio of rat red blood cells treated with CCMNRs at different concentrations (n = 3).


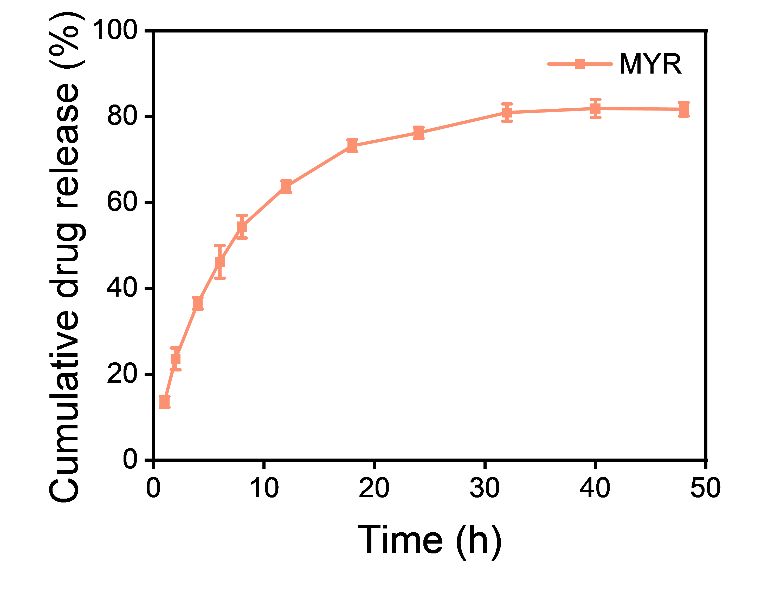


**Figure S10.** *In vitro* release profile of MYR from PBX@CCM hydrogel in PBS (10 mM, pH 7.4) at 37°C (n = 3).


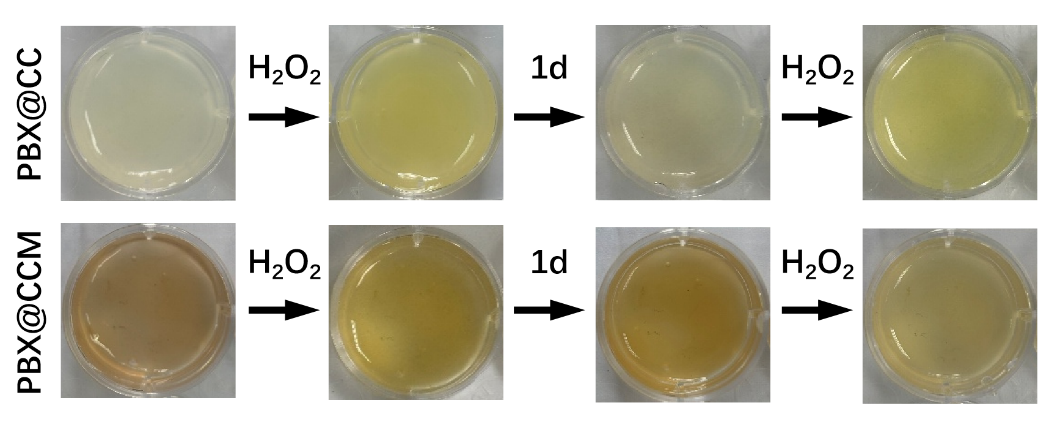


**Figure S11.** Digital photographs showing the color changes of PBX@CC and PBX@CCM hydrogels during cyclic 1 mM H_2_O_2_ treatment and subsequent recovery.


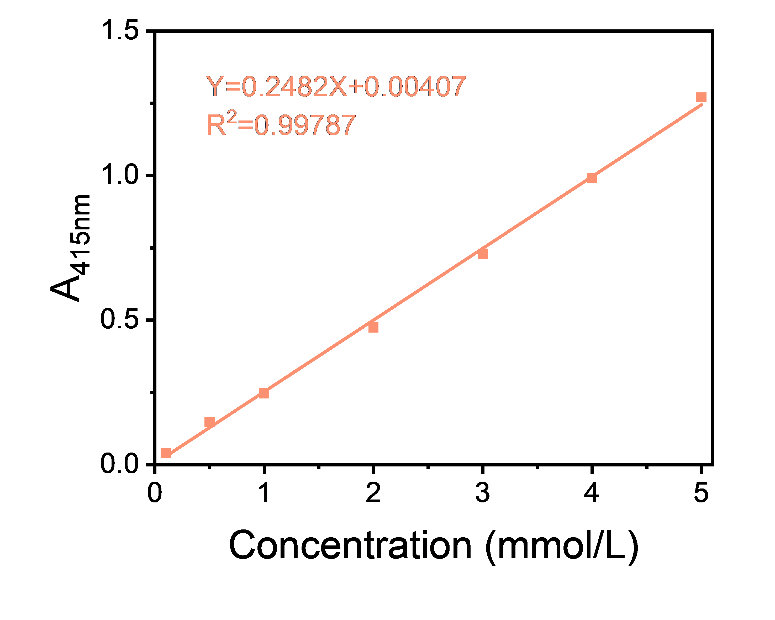


**Figure S12.** Standard calibration curve of titanium sulfate and hydrogen peroxide products by ultraviolet-visible spectroscopy.


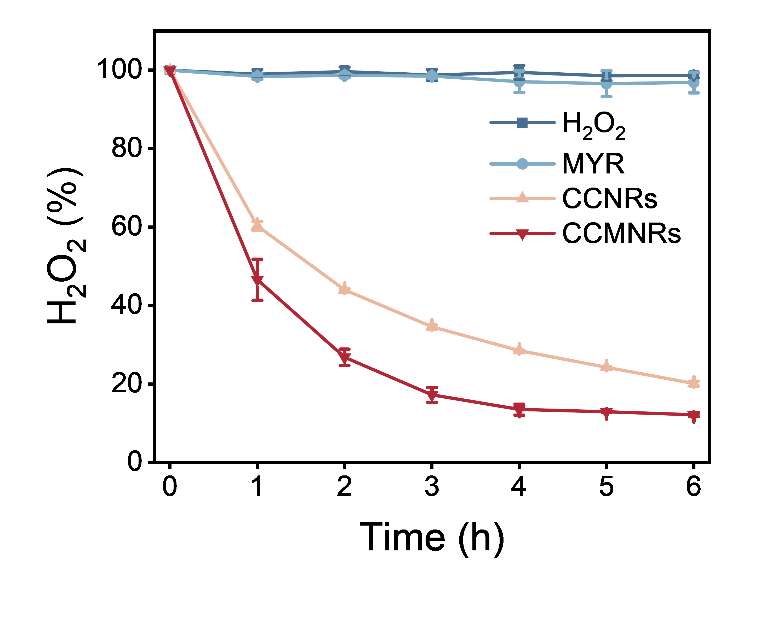


**Figure S13.** H_2_O_2_ scavenging activity of MYR, CCNRs, and CCMNRs in solution, measured using titanyl sulfate as the H_2_O_2_-specific probe (n = 3).


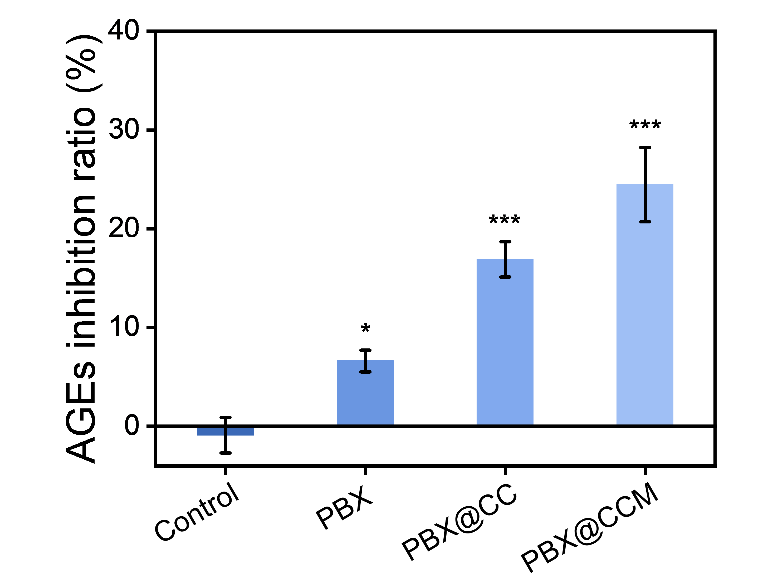


**Figure S14.** AGEs inhibition (%) of different treatments after 5 days (n = 3). * and *** indicate *p* < 0.05 and *p* < 0.001 compared with the control group, respectively.

**
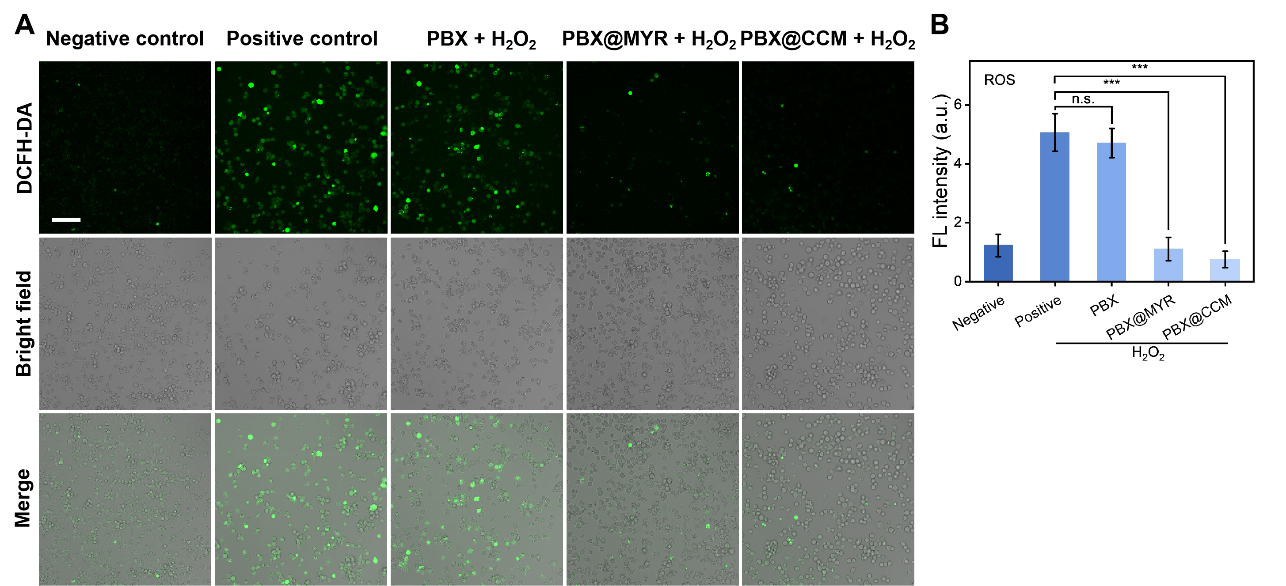
Figure S15.** Intracellular ROS scavenging ability of PBX@CCM hydrogel under H_2_O_2_ stimulation. (A) Representative confocal fluorescence images showing ROS expression in RAW 264.7 cells treated with different hydrogel extracts, using DCFH-DA probes to assess ROS levels. Scale bar = 100 μm. (B) Quantitative results of ROS fluorescence intensity (n = 3).
